# Altering the rate-determining step over cobalt single clusters leading to highly efficient ammonia synthesis

**DOI:** 10.1093/nsr/nwaa136

**Published:** 2020-06-17

**Authors:** Sisi Liu, Mengfan Wang, Haoqing Ji, Xiaowei Shen, Chenglin Yan, Tao Qian

**Affiliations:** College of Energy, Key Laboratory of Advanced Carbon Materials and Wearable Energy Technologies of Jiangsu Province, Soochow University, Suzhou 215006, China; College of Energy, Key Laboratory of Advanced Carbon Materials and Wearable Energy Technologies of Jiangsu Province, Soochow University, Suzhou 215006, China; College of Energy, Key Laboratory of Advanced Carbon Materials and Wearable Energy Technologies of Jiangsu Province, Soochow University, Suzhou 215006, China; College of Energy, Key Laboratory of Advanced Carbon Materials and Wearable Energy Technologies of Jiangsu Province, Soochow University, Suzhou 215006, China; College of Energy, Key Laboratory of Advanced Carbon Materials and Wearable Energy Technologies of Jiangsu Province, Soochow University, Suzhou 215006, China; College of Energy, Key Laboratory of Advanced Carbon Materials and Wearable Energy Technologies of Jiangsu Province, Soochow University, Suzhou 215006, China

**Keywords:** nitrogen reduction reaction, rate-determining step, cobalt single cluster, mass transfer, molecular dynamics simulations

## Abstract

Activation of high-energy triple-bonds of N_2_ is the most significant bottleneck of ammonia synthesis under ambient conditions. Here, by importing cobalt single clusters as strong electron-donating promoter into the catalyst, the rate-determining step of ammonia synthesis is altered to the subsequent proton addition so that the barrier of N_2_ dissociation can be successfully overcome. As revealed by density functional theory calculations, the N_2_ dissociation becomes exothermic over the cobalt single cluster upon the strong electron backdonation from metal to the N_2_ antibonding orbitals. The energy barrier of the positively shifted rate-determining step is also greatly reduced. At the same time, advanced sampling molecular dynamics simulations indicate a barrier-less process of the N_2_ approaching the active sites that greatly facilitates the mass transfer. With suitable thermodynamic and dynamic property, a high ammonia yield rate of 76.2 μg h^–1^ mg$^{-1 }_{\rm cat.}$ and superior Faradaic efficiency of 52.9% were simultaneously achieved.

## Teaser

The most significant bottleneck of ambient ammonia synthesis, namely nitrogen activation, has been successfully overcome by altering the rate-determining step over cobalt single clusters-contained electrocatalyst, and superior nitrogen reduction performance was achieved.

## INTRODUCTION

Ammonia (NH_3_) is widely considered as a critical chemical whether in agriculture or transportation [1,2], since it is the main ingredient for fertilizer production and a carbon-free energy storage intermediate with high-energy density [3]. Although an infinite nitrogen (N_2_) source from the atmosphere can be easily obtained, large scale ammonia production is hindered by the chemical stability of the N≡N bond (bond energy: 940.95 kJ mol^−1^) [4,5]. To date, the traditional Haber-Bosch process using transition metal as catalyst under drastic reaction conditions still dominates the industrial market of NH_3_ synthesis [6–8]. However, this typical strategy can only reach a relatively low conversion ratio (∼15%) and consumes nearly 5% of the world's natural gas [9,10]. The use of fossil fuels, at the same time, accounts for large quantities of CO_2_ generation into the atmosphere [11]. Therefore, a clean and sustainable strategy for NH_3_ production is urgently demanded for both the global population and energy.

The electrocatalytic N_2_ reduction reaction (NRR), using protons from water as the hydrogen source and powered by renewable electricity sources, is an alternative method to achieving N_2_ fixation under ambient conditions [12–16]. Theoretically, common mechanisms of the NRR start with N_2_ chemisorption on the catalyst’s surface, followed by the cleavage of N≡N bond and consecutive proton addition to form NH_3_ [17]. Yet, strong bonding energy, high ionization potential, broad HOMO-LUMO gap as well as poor electron affinity of N_2_ do not favor any electron transfer process, and the N_2_ activation process is thus commonly considered as the rate-determining step [18,19]. A catalyst which features active sites with suitable energy and symmetry of orbitals is able to bind with N_2_ molecules through accepting electron density from, and backdonating to, N_2_ [20]. The backdonation, known as the π backbonding, strengthens the catalyst–nitrogen bond, weakens the N≡N bond, and thus contributes to lowering the energy barrier of N_2_ activation and positively shifting the rate-determining step [19]. This process could be enhanced by strong electron-donating ability, which enables the smooth electron transfer from the active site to the N_2_ antibonding π-orbitals, termed the electronic promoting effect [21–23], and further benefits the eventual N_2_ dissociation. Unfortunately, only very few catalysts reported to date can efficiently reduce the nitrogen activation barrier, leaving the ammonia production rate and Faradaic efficiency in low level [23–25]. Hence, searching for highly active catalysts that could alter the rate-determining step of electrochemical ammonia synthesis is still a challenging goal.

**Figure 1.**
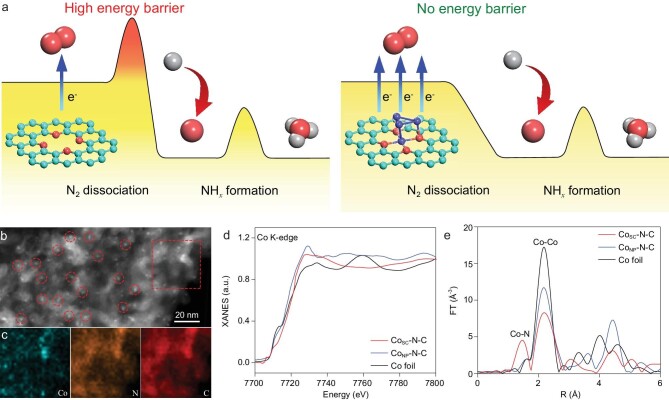
(a) Schematic illustration for the mechanism of enhanced NRR activity by introducing Co single cluster in nitrogen-doped carbon. The cyan, red, purple and gray spheres represent C, N, Co and H atoms, respectively. (b) Dark-field TEM image of Co_SC_-N-C showing highly dispersed Co single clusters in the material and (c) corresponding element maps showing the distribution of Co (blue), N (orange) and C (red). (d) Co K-edge X-ray absorption near-edge structure (XANES) and (e) Fourier-transformed (FT) k^3^-weighted extended X-ray absorption fine structure (EXAFS) spectra of Co_SC_-N-C, Co_NP_-N-C and Co foil.

Herein, we successfully demonstrate that deliberately introducing cobalt single clusters as electron-donating promoter in nitrogen-doped carbon alters the rate-determining step of ammonia synthesis from N_2_ cleavage to proton addition (Fig. 1a). An excellent ammonia yield rate (76.2 μg h^–1^ mg$^{-1 }_{\rm cat.}$) and a superior Faradaic efficiency (52.9%) were simultaneously obtained under ambient conditions. Isotopic labeling experiments and control experiments are combined to confirm that all the NH_3_ produced is from the N_2_ electrochemical reduction. Also, the catalyst is steady enough to suffer consecutive electrolysis recycle with negligible attenuation in the NRR activity and selectivity. Density functional theory (DFT) calculations reveal that N_2_ activation is transferred into a strong

thermodynamically exothermic process on cobalt single clusters, so that it is no longer the rate-determining step of ammonia synthesis. Instead, only small energy barriers exist upon NH*_x_* formation in the whole nitrogen fixation process. Also, the approaching process of N_2_ molecules towards the single cluster sites is confirmed to be barrier-less by molecular dynamics (MD) simulations, which greatly favors the whole nitrogen reduction process. Altering the rate-determining step of nitrogen reduction effectively leads to a desirable NRR performance, and thus provides a powerful guidance for future design of catalysts.

## RESULTS AND DISCUSSION

### Characterization of the Co_SC_-N-C catalyst

The cobalt single clusters dispersed in nitrogen-doped carbon (Co_SC_-N-C) were fabricated and carefully characterized. The transmission electron microscopy (TEM) image (Supplementary Fig. 1) and dark-field TEM image (Fig. 1b) of Co_SC_-N-C clearly show highly dispersed cobalt single clusters in the catalyst, with corresponding element mapping (Fig. 1c) demonstrating the uniform distribution of Co superimposing with C and N. Using aberration corrected high-angle annular dark-field scanning transmission electron microscopy (HAADF-STEM) with sub angstrom resolution, the clusters are observed to be of small size with an average diameter of approximately 0.5 nm (Supplementary Fig. 2). The overall Co content in the Co_SC_-N-C is about 3.15 wt%, as determined by inductively coupled plasma optical emission spectrometry (ICP-OES) analysis. To highlight the specific role of cobalt single clusters, counterparts with cobalt nanoparticles (Co_NP_-N-C) and without metal (N-C) were also synthesized by replacing the Co^2+^/Zn^2+^ mixture with single Co^2+^ and Zn^2+^, respectively, under otherwise identical conditions (Supplementary Figs 3 and 4). As shown in the X-ray powder diffraction (XRD) patterns (Supplementary Fig. 5), Co_NP_-N-C exhibits distinct metallic cobalt diffraction, whereas the cobalt single clusters in Co_SC_-N-C exist as amorphous phase. The increased content of Co accounts for the improved graphitization degree, as evaluated by the Raman spectra (Supplementary Fig. 6).

To confirm the chemical state of Co species in different samples, X-ray absorption fine structure (XAFS) measurements were conducted with Co foil as reference. The Co K-edge X-ray absorption near-edge structure (XANES) shows that the absorption edge of Co_SC_-N-C exhibits a positive shift compared with that of Co foil, reflecting that the average valence state of Co atoms is at positive level and is higher than that in Co_NP_-N-C (Fig. 1d). The Fourier-transformed (FT) k^3^-weighted extended X-ray absorption fine structure (EXAFS) spectrum of the Co_SC_-N-C (Fig. 1e) shows two main peaks at about 1.5 Å and 2.2 Å, attributing to Co-N and Co-Co, respectively. In great contrast, only a strong Co-Co coordination is detected in Co_NP_-N-C, so that the Co atoms are present as nanoparticles in the carbon framework. The surface chemistry of different samples was further investigated by X-ray photoelectron spectroscopy (XPS, Supplementary Fig. 7). The coexistence of four different N species in different samples, namely pyridinic-N, pyrrolic-N, graphitic-N and N-oxides, was confirmed by the high-resolution N 1s spectra (Supplementary Fig. 8) [26,27]. The high-resolution Co 2p spectra (Supplementary Fig. 9) show higher valence states of Co in Co_SC_-N-C compared with Co_NP_-N-C [28,29], indicating the coordination of cobalt and nitrogen, which is in accordance with the XAFS responses. The XPS results and the XAFS responses are combined to confirm the existence of nitrogen-stabilized cobalt single clusters in Co_SC_-N-C. In addition, Co_SC_-N-C possesses a large surface area of 287.1 m^2^ g^−1^, as determined by the Brunauer-Emmett-Teller (BET) method (Supplementary Fig. 10).

### Electroreduction of N_2_ to NH_3_ on the Co_SC_-N-C catalyst

**Figure 2.**
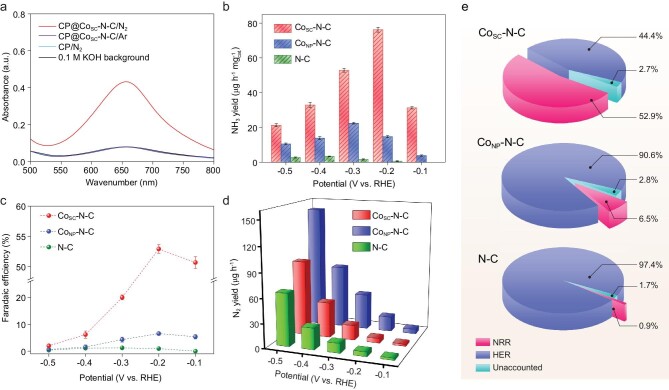
(a) The UV-vis absorption spectra of the electrolytes after electrolysis under different conditions. (b) NH_3_ yield rates and (c) corresponding Faradaic efficiencies at each given potential of Co_SC_-N-C, Co_NP_-N-C and N-C. (d) H_2_ yield of Co_SC_-N-C, Co_NP_-N-C and N-C at different potentials. The error bars correspond to the standard deviations of the obtained data over three separately conducted electrochemical measurements under the same conditions. (e) Selectivity of NRR and HER at −0.2 V vs. RHE of different samples.

To evaluate the NRR activity of different samples, linear sweep voltammetry (LSV) curves were first measured (Supplementary Fig. 11). The current density difference between Ar and N_2_ clearly affirms the contribution from the nitrogen reduction. Without cobalt sites, N-C exhibits the weakest NRR and hydrogen evolution reaction (HER) activity. While, compared with Co_NP_-N-C, the bigger current density gap between Ar and N_2_ and lower HER current density of Co_SC_-N-C indicate that the introduction of cobalt single clusters leads to much more improved NRR activty and selectivity than those of the cobalt nanoparticles. Then, a quantified study of the NRR ability for different samples was carried out via chronoamperometry measurement using an H-shape electrochemical cell [30]. Here, the most rigorous experimental protocol was followed for reliable proof of the NRR performance [31]. The nitrogen gas was sufficiently purified before use to avoid the possible existence of NH_3_ and NO_x_. Possible reduction products including NH_3_ and N_2_H_4_ were both tested (Supplementary Figs 12–15), whereas only NH_3_ was detected in this work (Supplementary Fig. 16). Before the NRR test, several control experiments were first carried out to ensure that no contamination was present in the feeding gas or the equipment, and the carbon paper (CP) as current collector of the cathode did not have NRR activity, so that NH_3_ could only be produced by N_2_ reduction in the presence of catalyst (Fig. 2a). Detailed comparison of the NH_3_ yield rates and corresponding Faradaic efficiencies of Co_SC_-N-C, Co_NP_-N-C and N-C under various applied potentials is displayed in Fig. 2b and c. Clearly, pristine N-C exhibits only negligible NRR performance. After incorporating cobalt single clusters, the Co_SC_-N-C, with an optimized loading of 0.5 mg cm^–2^ (Supplementary Fig. 17), exhibits the highest NH_3_ yield rate of 76.2 μg h^–1^ mg$^{-1 }_{\rm cat.}$ at −0.2 V versus reversible hydrogen electrode (vs. RHE), far exceeding the peak value of Co_NP_-N-C (22.5 μg h^−1^ mg$^{-1 }_{\rm cat.}$) realized at an even more negative potential (−0.3 V vs. RHE). Notably, the maximal NRR Faradaic efficiency of Co_SC_-N-C is also obtained at −0.2 V vs. RHE (52.9%), which is more than one order of magnitude higher than the counterparts. Detailed chronoamperometry responses are shown in Supplementary Fig. 18. Also, the electrochemical active surface area (EASA) of each sample was determined by double-layer capacitance (C_dl_, Supplementary Fig. 19). Expectedly, the surface-area-normalized ammonia production rate of Co_SC_-N-C shows an obvious advantage over other samples (Supplementary Fig. 20). The NRR activity and selectivity of Co_SC_-N-C remarkably stands at the top level of reported catalysts under mild conditions (Supplementary Table 1), and is thus of great significance in energy utilization. Based on the nitrogen temperature-programmed desorption (N_2_-TPD) spectra (Supplementary Fig. 21), Co_SC_-N-C exhibits the strongest N_2_ adsorption ability, confirming that cobalt single clusters are superior nitrogen-adsorption active sites compared with cobalt nanoparticles or nitrogen-doped carbon [32]. The gradual decrease of the NRR Faradaic efficiency of Co_SC_-N-C as the applied potential becomes more negative than −0.2 V vs. RHE is mainly due to the increased HER [33], as evaluated by the gas chromatography (GC) responses (Fig. 2d and Supplementary Fig. 22). In terms of selectivity, Co_SC_-N-C shows a much smaller proportion of the competing HER compared with Co_NP_-N-C and N-C, and is more inclined to proceed NRR (Supplementary Fig. 23). Based on the above results, Co_SC_-N-C has a prominent advantage in nitrogen reduction, especially at −0.2 V vs. RHE (Fig. 2e and Supplementary Table 2).

**Figure 3.**
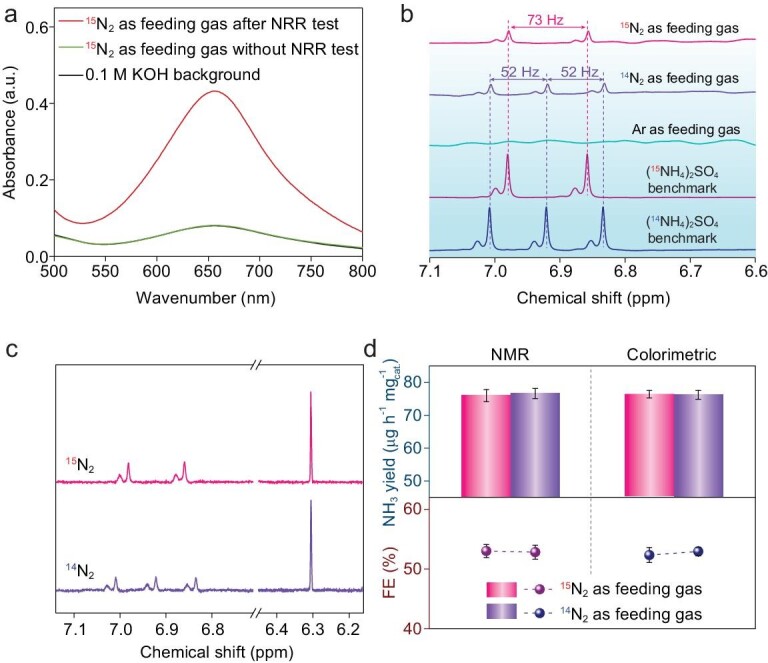
(a) The UV-vis absorption spectra of the electrolytes after electrolysis using ^15^N_2_ as feeding gas under different conditions. (b) ^1^H nuclear magnetic resonance (NMR) spectra of both ^14^NH_4_^+^ and ^15^NH_4_^+^ produced from the NRR using ^14^N_2_ and ^15^N_2_ as feeding gas, respectively. (c) ^1^H NMR spectra of the NRR product at −0.2 V vs. RHE using NMR quantification method. (d) Comparison of NH_3_ yield rate and Faradaic efficiency for NRR at −0.2 V vs. RHE using different quantification methods. The error bars correspond to the standard deviations of the obtained data over three separately conducted electrochemical measurements under the same conditions.

Careful examination of the N source for produced NH_3_ is helpful in getting an in-depth understanding of the catalyzing mechanism. Thus, isotope-labeling experiments were systematically conducted [34–37]. The ^15^N_2_ gas was also sufficiently purified before use. As shown in the ultraviolet-visible (UV–vis) absorption spectra (Fig. 3a), no NH_3_ can be detected when ^15^N_2_ is fed unless an electrocatalytic potential was applied to the Co_SC_-N-C working electrode. Then, the produced NH_3_ was distinguished using ^1^H nuclear magnetic resonance (^1^H NMR) spectra (Fig. 3b). After a continuous electrolysis under −0.2 V vs. RHE using ^15^N_2_ as feeding gas, only a doublet signal (∼73 Hz) representing ^15^NH_4_^+^ is detected in the spectra instead of the triplet signal (∼52 Hz) of ^14^NH_4_^+^. When Ar was used for electrolysis, no NH_3_ signal was detected in the NMR spectrum, indicating the obtained ammonia is totally from the N_2_ electroreduction process. For accuracy of the performance data, NMR method was also employed for ammonia quantification (Fig. 3c and Supplementary Fig. 24). The NH_3_ yield rate and Faradaic efficiency obtained by quantitative ^1^H NMR and colorimetric method using both ^14^N_2_ and ^15^N_2_ exhibit good consistency (Fig. 3d), thus demonstrating the reliability of the experimental results.

Stability is another vital parameter for an electrocatalyst. Thus, the durability of Co_SC_-N-C was consecutively tested by electrolyzing at a constant potential of −0.2 V vs. RHE for 10 cycles. The Co_SC_-N-C catalyst can keep the superior Faradaic efficiency of NH_3_ production unchanged even after 10 cycles of continuous electrolysis (Supplementary Fig. 25), manifesting its broad prospect for practical applications. Simultaneously, TEM image, corresponding element maps and Raman spectra (Supplementary Figs 26 and 27) exhibit no variation in the morphology and structural properties of Co_SC_-N-C after the NRR process. Its chemical state is also well maintained as confirmed by the high-resolution XPS spectra analyses (Supplementary Fig. 28), demonstrating that the Co_SC_-N-C is robust enough for long-term NRR electrocatalysis.

### Computational studies

**Figure 4.**
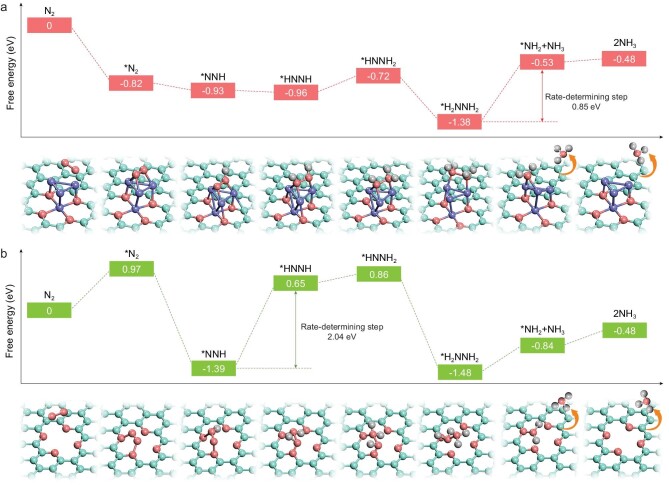
Free energy diagram and models represent the corresponding adsorbates on (a) Co_4_-N_4_/C and (b) N_4_/C through the associative alternating pathway. The cyan, red, purple and gray spheres represent C, N, Co and H atoms, respectively.

**Figure 5.**
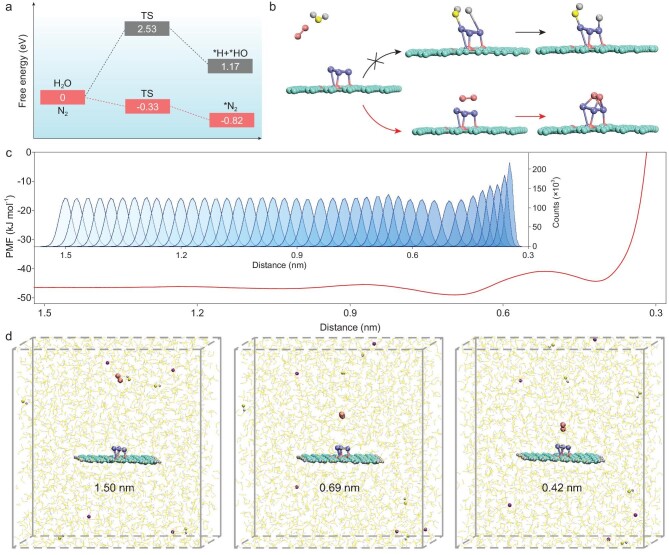
(a) Calculated free energy and (b) computational models of the transition state (TS) of hydrogen and nitrogen chemisorption on Co_4_-N_4_/C model. The cyan, red, purple, yellow and gray spheres represent C, N, Co, O and H atoms, respectively. (c) PMF for N_2_ adsorption on the Co_4_-N_4_ model in 0.1 M KOH; inset: complete histograms of all window umbrella sampling statistics used for calculation of the N_2_ adsorption on the Co_4_-N_4_ model. (d) Major part of the MD simulation snapshots at 1.50 nm, 0.69 nm and 0.42 nm, with N_2_ (red spheres), H_2_O (yellow and gray sticks), OH^−^ (yellow and gray spheres) and K^+^ (wine red spheres).

Computational studies on both thermodynamics and dynamics were carried out to investigate the mechanism of ammonia synthesis over the Co_SC_-N-C catalyst. The thermodynamic process of different models was first studied by DFT calculations. As confirmed by catalyst characterizations, the cobalt in Co_SC_-N-C mainly exists as single clusters. Accordingly, several possible models of cobalt single cluster with different numbers of cobalt atoms on nitrogen-doped carbon (Co_x_-N_4_/C, x = 2 to 6) were systematically proposed, and pure nitrogen-doped carbon (N_4_/C) was also calculated for comparison (Supplementary Fig. 29). For N_4_/C, the nitrogen adsorption is a strong endothermic process with a step-by-step uphill trend of Gibbs free energy (Supplementary Fig. 30). The introduction of cobalt single clusters is able to turn the nitrogen adsorption into exothermic process without energy barriers, suggesting that the cobalt single clusters are superior nitrogen-adsorption active sites. Notably, all of the structures with cobalt single clusters are able to alter the rate-determining step to the subsequent nitrogen hydrogenation with relatively small energy barriers (Supplementary Figs 31 and 32). Taking Co_4_-N_4_/C for example, as clearly shown in Fig. 4a, starting from a favored side-on mode N_2_ adsorption, the preferred NRR approach of Co_4_-N_4_/C is verified to be the associative alternating pathway instead of the associative distal pathway (Supplementary Fig. 33a). When chemically adsorbed on Co cluster, N_2_ is spontaneously activated and experiences a significant weakening of the N≡N bond due to the strong electron backdonation from the metal to the N_2_ antibonding orbitals, and the N_2_ dissociation becomes an exothermic process over the cobalt single cluster. In addition, the energy released from the N_2_ adsorption step greatly benefits the following N_2_ cleavage, according to the ‘hot atom’ mechanism [19,38]. Thus, the rate-determining step has been successfully shifted from the usual N_2_ activation to the subsequent hydrogenation with only a small energy barrier of 0.85 eV. In great contrast, without the Co single cluster, the N_4_/C model not only suffers a severely endothermic N_2_ adsorption process, but also possesses high NRR rate-limiting barriers of 2.04 eV and 1.84 eV for the alternating and distal pathways, respectively, indicating a weak activity towards NRR (Fig. 4b and Supplementary Fig. 33b). On the other hand, N_2_ has priority in the adsorption competition with ^*^H on Co_4_-N_4_/C. As shown in Fig. 5a and b, the N_2_ chemisorption on Co_4_-N_4_/C is strongly exothermic (−0.82 eV) with a step-by-step downhill trend of the Gibbs free energy, whereas the H chemisorption suffers an extremely high energy barrier of 2.53 eV due to the water dissociation process. Even if ^*^H is adsorbed, its desorption to form H_2_ is still an endothermic reaction (Supplementary Fig. 34), which is also beneficial to promoting the NRR Faradaic efficiency.

From a dynamic point of view, the N_2_ approaching process towards Co_4_-N_4_/C was then explored by advanced sampling MD simulations. The system was set up by randomly placing 6 K^+^, 6 OH^−^ and 3000 water molecules in the simulation box, with a Co_4_-N_4_/C model fixed perpendicular to the z-axis at the center of the simulation system. The N_2_ molecule initially located at 1.5 nm above the geometric center of the upper three Co atoms. Then, the N_2_ molecule was pulled towards the Co_4_-N_4_/C along the z-axis at a rate of 0.01 nm ps^–1^ under a harmonic force constant to generate configurations for umbrella-sampling (Fig. 5c). The potential of the mean force (PMF) as a function of distance and the corresponding snapshots (Fig. 5d and Supplementary Fig. 35) illustrate that the approaching process of N_2_ molecule is nearly barrier-less with only a small energy hill at approximately 0.52 nm. This clearly indicates that the cobalt single cluster as active site is accessible to the N_2_ molecule, which can greatly promote the following N_2_ chemisorption, and is thus contributory to the entire NRR process. The above computational conclusions well explain the experimental

results, and further highlight the contribution of the cobalt single cluster as an important promoter for ambient N_2_ fixation.

## CONCLUSION

In summary, we have successfully altered the rate-determining step of ambient NH_3_ synthesis by deliberate introduction of cobalt single clusters as electron-donating promoter in nitrogen-doped carbon, and achieved outstanding ammonia yield rate of 76.2 μg h^–1^ mg$^{-1 }_{\rm cat.}$ and superior Faradaic efficiency of 52.9%. The Co_SC_-N-C is steady enough for long-term NRR electrocatalysis with negligible decay of the amazingly high Faradaic efficiency through consecutive electrolysis recycle. The ^15^N isotopic labeling experiments and control experiments are combined to confirm that the N source of N_2_-to-NH_3_ conversion is completely from the feeding gas instead of any activated N species in the catalyst. First-principles simulations demonstrate a strong exothermic process of N_2_ chemisorption on the cobalt single cluster site, which greatly promotes the following N_2_ dissociation. The N_2_ activation, therefore, becomes exothermic, and the rate-determining step has been successfully altered to the subsequent nitrogen hydrogenation. A smooth N_2_ approaching process with almost no energy hill towards the catalyst paves the way for the N_2_ mass transfer, as confirmed by the MD simulations. Our work overcomes the obstacle of ambient ammonia synthesis, and contributes to the guiding of future catalyst design for sustainable NRR systems.

## Supplementary Material

nwaa136_Supplemental_File

## References

[1] Gruber N , Galloway JN. An earth-system perspective of the global nitrogen cycle. Nature 2018; 451: 293–6. 10.1038/nature06592 18202647

[2] Li X , Li T, Ma Y et al. Boosted electrocatalytic N_2_ reduction to NH_3_ by defect-rich MoS_2_ nanoflower. Adv Energy Mater 2018; 8: 1801357.

[3] Ali M , Zhou F, Chen K et al. Nanostructured photoelectrochemical solar cell for nitrogen reduction using plasmon-enhanced black silicon. Nat Commun 2016; 7: 11335. 27093916 10.1038/ncomms11335 PMC4842983

[4] van der Ham CJM , Koper MTM, Hetterscheid DGH. Challenges in reduction of dinitrogen by proton and electron transfer. Chem Soc Rev 2014; 43: 5183–91. 24802308 10.1039/c4cs00085d

[5] Shi M-M , Bao D, Wulan B-R et al. Au sub-nanoclusters on TiO_2_ toward highly efficient and selective electrocatalyst for N_2_ conversion to NH_3_ at ambient conditions. Adv Mater 2017; 29: 1606550. 10.1002/adma.201606550 28240391

[6] Licht S , Cui B, Wang B et al. Ammonia synthesis by N_2_ and steam electrolysis in molten hydroxide suspensions of nanoscale Fe_2_O_3_. Science 2014; 345: 637–40. 25104378 10.1126/science.1254234

[7] Wang M , Liu S, Qian T et al. Over 56.55% Faradaic efficiency of ambient ammonia synthesis enabled by positively shifting the reaction potential. Nat Commun 2019; 10: 341. 30664636 10.1038/s41467-018-08120-x PMC6341113

[8] Wang L , Xia M, Wang H et al. Greening ammonia toward the solar ammonia refinery. Joule 2018; 2:1055–74.

[9] Wang J , Yu L, Hu L et al. Ambient ammonia synthesis via palladium-catalyzed electrohydrogenation of dinitrogen at low overpotential. Nat Commun 2018; 9: 1795. 29765053 10.1038/s41467-018-04213-9 PMC5953946

[10] Li SJ , Bao D, Shi M-M et al. Amorphizing of Au nanoparticles by CeO_x_-RGO hybrid support towards highly efficient electrocatalyst for N_2_ reduction under ambient conditions. Adv Mater 2017; 29: 1700001. 10.1002/adma.201700001 28681965

[11] Zhang L , Ji X, Ren X et al. Electrochemical ammonia synthesis via nitrogen reduction reaction on a MoS_2_ catalyst: theoretical and experimental studies. Adv Mater 2019; 30: 1800191. 10.1002/adma.201800191 29808517

[12] Seh ZW , Kibsgaard J, Dickens CF et al. Combining theory and experiment in electrocatalysis: insights into materials design. Science 2017; 355: eaad4998. 28082532 10.1126/science.aad4998

[13] Lv C , Yan C, Chen G et al. An amorphous noble-metal-free electrocatalyst enables N_2_ fixation under ambient conditions. Angew Chem Int Ed 2018; 57: 6073–6. 10.1002/anie.201801538 29473991

[14] Liu S , Wang M, Qian T et al. Facilitating nitrogen accessibility to boron-rich covalent organic frameworks via electrochemical excitation for efficient nitrogen fixation. Nat Commun 2019; 10: 3898. 31467283 10.1038/s41467-019-11846-x PMC6715660

[15] Chen G-F , Ren S, Zhang L et al. Advances in electrocatalytic N_2_ reduction-strategies to tackle the selectivity challenge. Small Methods 2019; 3: 1800337.

[16] Qiu W , Xie XY, Qiu J et al. High-performance artificial nitrogen fixation at ambient conditions using a metal-free electrocatalyst. Nat Commun 2018; 9: 3485. 30154483 10.1038/s41467-018-05758-5 PMC6113289

[17] Guo C , Ran J, Vasileff A et al. Rational design of electrocatalysts and photo(electro)catalysts for nitrogen reduction to ammonia (NH_3_) under ambient conditions. Energy Environ Sci 2018; 11: 45–56.

[18] Cui X , Tang C, Zhang Q. A review of electrocatalytic reduction of dinitrogen to ammonia under ambient conditions. Adv Energy Mater 2018; 8: 1800369.

[19] Gong Y , Wu J, Kitano M et al. Ternary intermetallic LaCoSi as a catalyst for N_2_ activation. Nat Catal 2018; 1: 178–85.

[20] Légaré M-A , Bélanger-Chabot G, Dewhurst RD et al. Nitrogen fixation and reduction at boron. Science 2018; 359: 896–900. 29472479 10.1126/science.aaq1684

[21] Ma X-L , Liu J-C, Xiao H et al. Surface single-cluster catalyst for N_2_-to-NH_3_ thermal conversion. J Am Chem Soc 2018; 140: 46–9. 29244491 10.1021/jacs.7b10354

[22] Kitano M , Inoue Y, Yamazaki Y et al. Ammonia synthesis using a stable electride as an electron donor and reversible hydrogen store. Nat Chem 2012; 4: 934–40. 23089869 10.1038/nchem.1476

[23] Kitano M , Kanbara S, Inoue Y et al. Electride support boosts nitrogen dissociation over ruthenium catalyst and shifts the bottleneck in ammonia synthesis. Nat Commun 2015; 6: 6731. 25816758 10.1038/ncomms7731 PMC4389256

[24] Wang P , Chang F, Gao W et al. Breaking scaling relations to achieve low-temperature ammonia synthesis through LiH-mediated nitrogen transfer and hydrogenation. Nat Chem 2017; 9: 64–70. 27995914 10.1038/nchem.2595

[25] Inoue Y , Kitano M, Kishida K et al. Efficient and stable ammonia synthesis by self-organized flat Ru nanoparticles on calcium amide. ACS Catal 2016; 6: 7577–84.

[26] Fei H , Dong J, Arellano-Jiménez A et al. Atomic cobalt on nitrogen-doped graphene for hydrogen generation. Nat Commun 2015; 6: 8668. 26487368 10.1038/ncomms9668 PMC4639894

[27] Fu J , Hassan FM, Li J et al. Flexible rechargeable zinc-air batteries through morphological emulation of human hair array. Adv Mater 2016; 28: 6421–8. 27197721 10.1002/adma.201600762

[28] Jin H , Wang J, Su D et al. In situ cobalt−cobalt oxide/N-doped carbon hybrids as superior bifunctional electrocatalysts for hydrogen and oxygen evolution. J Am Chem Soc 2015; 137: 2688–94. 25658518 10.1021/ja5127165

[29] Guan C , Sumboja A, Zang W et al. Decorating Co/CoN_x_ nanoparticles in nitrogen-doped carbon nanoarrays for flexible and rechargeable zinc-air batteries. Energy Storage Mater 2019; 16: 243–50.

[30] Bao D , Zhang Q, Meng F-L et al. Electrochemical reduction of N_2_ under ambient conditions for artificial N_2_ fixation and renewable energy storage using N_2_/NH_3_ cycle. Adv Mater 2017; 29: 1604799. 10.1002/adma.201604799 27859722

[31] Andersen SZ , Čolić V, Yang S et al. A rigorous electrochemical ammonia synthesis protocol with quantitative isotope measurements. Nature 2019; 570: 504–8. 31117118 10.1038/s41586-019-1260-x

[32] Zhang L , Ding L-X, Chen G-F et al. Ammonia synthesis under ambient conditions: selective electroreduction of dinitrogen to ammonia on black phosphorus nanosheets. Angew Chem Int Ed 2019; 58: 2612–6. 10.1002/anie.201813174 30560583

[33] Chen H , Ding L-X, Chen G-F et al. Molybdenum carbide nanodots enable efficient electrocatalytic nitrogen fixation under ambient conditions. Adv Mater 2018; 30: 1803694. 10.1002/adma.201803694 30276883

[34] Chen H , Cui P, Wang F et al. High efficiency electrochemical nitrogen fixation achieved on a low-pressure reaction system by changing chemical equilibrium. Angew Chem Int Ed 2019; 58: 15541–7. 10.1002/anie.201910658 31502747

[35] Luo Y , Chen G-F, Ding L et al. Efficient electrocatalytic N_2_ fixation with MXene under ambient conditions. Joule 2019; 3: 279–89.

[36] Chen G-F , Cao X, Wu S et al. Ammonia electrosynthesis with high selectivity under ambient conditions via a Li^+^ incorporation strategy. J Am Chem Soc 2017; 139: 9771–4. 28693318 10.1021/jacs.7b04393

[37] Zhou F , Azofra LM, Ali M et al. Electro-synthesis of ammonia from nitrogen at ambient temperature and pressure in ionic liquids. Energy Environ Sci 2017; 10: 2516–20.

[38] Wang J , Zhang L, Zeng Q et al. Adsorption of atomic and molecular oxygen on 3C-SiC(111) and (111) surfaces: a first-principles study. Phys Rev B 2009; 79: 125304.

