# Supplementary material for: Altering the rate-determining step over cobalt single clusters leading to highly efficient ammonia synthesis
Source: Natl Sci Rev. 2020 Jun 17;8(5):nwaa136. doi: 10.1093/nsr/nwaa136 (PMC8288418; doi:10.1093/nsr/nwaa136)
Supplement: nwaa136_Supplemental_File [file nwaa136_supplemental_file.docx]

**Supplementary Information**

**Altering the rate-determining step over cobalt single clusters leading to highly efficient ammonia synthesis**

Sisi Liu, Mengfan Wang, Haoqing Ji, Xiaowei Shen, Chenglin Yan & Tao Qian*

College of Energy, Key Laboratory of Advanced Carbon Materials and Wearable Energy Technologies of Jiangsu Province, Soochow University, Suzhou 215006, China. *Correspondence to: T.Q. (email: tqian@suda.edu.cn).

**METHODS**

**Catalyst** **synthesis.** N-doped carbon nanosheet substrate was first prepared. 2 ml pyrrole was adequately dispersed in sodium chloride solution by ultrasonic vibration. 1 ml 1 M HCl was added into the above solution, followed by dropwise adding of (NH_4_)_2_S_2_O_8_ solution into the system under vigorous stirring to start the pyrrole polymerization. The reaction was kept in ice/water bath for 24 h, and the obtained solution was freeze-dried. The obtained material was grinded into powders and carbonized at 900 °C for 2 h under argon atmosphere. After removing the salt template and impurities through sufficient washing with hydrochloric acid and lots of water and ethanol, the samples were dried under vacuum at 60 °C overnight prior to use. To prepare Co_SC_-N-C, 150 mg of as-prepared N-doped substrates were uniformly dispersed into 40 mL of methanol containing 0.4 g of polyvinyl pyrrolidone. The addition of polyvinyl pyrrolidone is to ensure the uniform growth of metal organic frameworks on the substrate surface. Then, Co(NO_3_)_2_·6H_2_O and Zn(NO_3_)_2_·6H_2_O with a molar ratio of 1:4 were subsequently dissolved into the above solution and stirred for 2 h. Then, 2-methylimidazole dissolved in 40 mL of methanol was dropwise added into the above solution under vigorous stirring. The reaction was kept stirring for another 30 min. The products were collected by centrifugation, washed thoroughly with methanol for several times, and dried at 60 °C overnight. The obtained powders were carbonized at 900 °C for 2 h under argon protection and cooled down to room temperature naturally. Impurities were washed sufficiently with lots of water and ethanol. Finally, the black powders were dried under vacuum at 60 °C overnight. Co_NP_-N-C and N-C were synthesized using the same method by replacing the Co(NO_3_)_2_·6H_2_O/Zn(NO_3_)_2_·6H_2_O mixture with pure Co(NO_3_)_2_·6H_2_O and Zn(NO_3_)_2_·6H_2_O of same molar, respectively. It should be noticed that, zinc in the precursor has been completely evaporated during the pyrolysis process, and does not exist in the resulting catalysts.

**Physical** **characterization.** The morphology was studied via a field emission transmission electron microscope (FETEM, FEI Tecnai G2 F20 S-TWIN TMP, Hongkong). Surface elemental analysis was performed on XPS (Kratos Axis Ultra Dld, Japan). The cobalt concentrations of the samples were conducted on the inductively coupled plasma optical emission spectrometry (ICP-OES). The X-ray absorption find structure spectra (Co K-edge) were obtained at 1W1B station in Beijing Synchrotron Radiation Facility (BSRF). The data collection was carried out in transmission mode using Si (111) double-crystal monochromator, ionization chamber for Co foil and in fluorescence excitation mode using a Lytle detector for Co_SC_-N-C and Co_NP_-N-C. N_2_-sorption analysis was performed by an ASAP 2020 accelerated surface area and porosimetry instrument (Micromeritics), equipped with automated surface area, at 77 K using BET calculations for the surface area. The structure of the catalysts was characterized by Raman spectroscopy (HR evolution, Horiba Jobin Yvon, France). Nitrogen temperature-programmed desorption (N_2_-TPD) measurement was conducted on an Auto Chem II 2920 instrument.

**Nitrogen purification.** High purity ^14^N_2_ (Messer Gas, Germany) and ^15^N_2_ (99 atom % ^15^N, Newradar Special Gas Co. Ltd., Wuhan, China) was purchased commercially. The gas was successively flowed through acid and alkaline trap to remove the possible NH_3_ and NO_x_. Then, the nitrogen gas was passed through a drying tube to avoid the vapor before supplying into the cell. The purified gas was ensured that no NH_3_ or NO_x_ resided.

**Cathode preparation.** Typically, 2 mg catalyst and 10 μL of Nafion solution (5 wt%) were dispersed in 190 μL ethanol and sonicated for at least 1 h to form a homogeneous ink. 50 μL catalyst ink was loaded on carbon paper with area of 1 × 1 cm^2^ and dried in N_2_ atmosphere at 80 ^o^C for 1 h. The carbon paper was used after hydrophilic treatment.

**Electrochemical NRR measurements.** The reduction of N_2_ gas was performed in a two-compartment cell at room temperature, which was separated by Celgard membrane. Both cathode chamber and anode chamber contained 40 ml of 0.1 M KOH electrolyte. A conventional three-electrode system using a electrochemical workstation was used to conduct electrochemical measurements, with Co_SC_-N-C, Co_NP_-N-C or N-C as the working electrode, Ag/AgCl (4 M KCl) as the reference electrode, and graphite rod as the counter electrode. For electrochemical NRR, potentiostatic tests were conducted in N_2_-saturated 0.1 M KOH, which was purged with N_2_ for 30 min before the measurement. Pure N_2_ was continuously fed into the cathodic compartment with a properly positioned sparger to ensure the whole cathode was hit by exquisite N_2_ gas bubbles during the experiments. For comparison, potentiostatic tests in Ar-saturated 0.1 M KOH were also conducted in this work. The gas flow rate is 30 sccm for both nitrogen and argon.

The potentiostatic tests were tested in 0.1 M KOH aqueous solution at different potentials including -0.1 V, -0.2 V, -0.3 V, -0.4 V, and -0.5 V vs. RHE. In order to avoid the loss of produced NH_3_ by N_2_ blowing during the test, another glass tube filled with 40 ml of 0.001 M H_2_SO_4_ as the gas absorption liquid was set at the end of the cell. The total NH_3_ production yield was the summation of NH_3_ in 0.1 M KOH and 0.001 M H_2_SO_4_. After electrochemical reduction reaction, the electrolyte and gas absorption liquid were both collected and analysed by colorimetric method or NMR spectra for qualitative measurements.

**Detection of produced ammonia.** Concentration of produced ammonia in 0.1 M KOH was spectrophotometrically determined by the indophenol blue method. In detail, 2 ml of the electrolyte after NRR potentiostatic test was removed from the electrochemical reaction vessel. 2 ml of a 1 M NaOH solution containing sodium citrate and salicylic acid was added. Then, 1 ml of 0.05 M NaClO and 0.2 ml of 1 wt % C_5_FeN_6_Na_2_O (sodium nitroferricyanide) were also added into the above solution. After standing in darkness at room temperature for 3 hours, the UV-Vis absorption spectrum was measured. The concentration of indophenol blue was determined using the absorbance at a wavelength of 655 nm. The concentration-absorbance curves were calibrated using standard ammonium sulfate solution with a serious of concentrations in 0.1 M KOH. Concentration of produced ammonia in 0.001 M H_2_SO_4_ was determined using the same method. For the NMR measurement, the standard curve is constructed by measuring a series of area of NMR resonance peak for the reference solutions with different ammonium sulfate concentrations.

**Determination of produced hydrazine.** The hydrazine presented in 0.1 M KOH was estimated by the method of Watt and Chrisp. A mixture of para-(dimethylamino) benzaldehyde (5.99 g), HCl (concentrated, 30 ml) and ethanol (300 ml) was used as a color reagent. 5 ml of the residual electrolyte after NRR potentiostatic test was removed from the electrochemical reaction vessel. Then, 5 ml of above prepared color reagent was added to the solution and stirred for 10 min at room temperature. The absorbance of the resulting solution was measured at a wavelength of 455 nm. The concentration-absorbance curves were calibrated using standard hydrazine hydrate-nitrogen 0.1 M KOH solution with a serious of concentrations. Concentration of produced hydrazine in 0.001 M H_2_SO_4_ was determined using the same method.

**Determination of HER productions.** The H_2_ was manual sampling and analyzed by gas chromatography (GC, Agilent 7890B).

**Calculation of the equilibrium potential.** The NRR standard potential at alkaline medium was calculated from the standard Gibbs energy of formation at 298.15 K.

| N_2_ (g) + 8H_2_O (l) + 6e^−^ → 2NH_4_OH (aq) + 6OH^−^  ΔG^o^ = 426.38 kJ mol^−1^ | (1) |
| --- | --- |
| E^o^ = −ΔG^o^/nF = −0.737 vs. standard hydrogen electrode (SHE) | (2) |

where n is the number of transferred electrons (6) and F is the Faraday constant (96,485 C mol^−1^).

Assuming 1 atm of N_2_, the thermodynamic equilibrium potential under the reaction conditions was calculated according to the Nernst equation.

| E = E° − RT / 6F × ln[c^2^(NH_~~4~~_OH) × c^6^(OH^−^)] + 0.059 × pH (vs. RHE) | (3) |
| --- | --- |

where R is the gas constant (8.314 J mol^−1^ K^−1^), T is the temperature in Kelvin (298.15 K), F is the Faraday constant (96,485 C mol^−1^), c(OH^−^) is the hydroxide concentration (0.1 M), pH is 13. Assuming a NH_~~4~~_OH concentration of 10^−7^ M in the solution, the corresponding thermodynamic equilibrium potentials is determined to be 0.23 V vs. RHE.

**Faradaic efficiency and the yield rate.** The yield rate and Faradaic efficiency of NH_3_ were calculated as below:

|  | Faradaic efficiency(NH_3_) = [3F × c(NH_3_) × V] / Q | (4) |
| --- | --- | --- |
|  | Yield rate(NH_3_) = [17c(NH_3_) × V] / (t × m) | (5) |

where F is the Faraday constant (96,485 C mol^−1^), t is the electrolysis time (1 h), m is the loading mass of the catalysts, Q is the total charge passed through the electrode, V is the volume of the electrolyte, and c(NH_3_) is the measured ammonia concentration.

The electrochemical double-layer capacitance (C_dl_) of the materials was measured to determine their electrochemical active surface area (EASA) using the cyclic voltammograms (CVs) in a small potential range with no faradic processes between 0.9 to 1.0 V vs. RHE. The plotted current density against scan rate has a liner relationship and its slope is twice the C_dl_. The EASA can then be calculated as below:

|  | A_EASA_ = C_dl_ of catalyst (mF cm^−2^) / 40 μF cm^−2^ per cm_EASA_ | (6) |
| --- | --- | --- |

The surface-area-normalized activity of NH_3_ was calculated as below:

|  | Yield rate_EASA_(NH_3_) = [17c(NH_3_) × V] / (t × A_EASA_) | (7) |
| --- | --- | --- |

The Faradaic efficiency of H_2_ was calculated as below:

|  | Faradaic efficiency(H_2_) = 2Fv(H_2_)Gp_0_ / RT_0_i_total_ | (8) |
| --- | --- | --- |

where v(H_2_) is the volume concentration of H_2_ in the exhaust gas from the electrochemical cell (GC data), G is the gas flow rate (ml/min at room temperature and ambient pressure), i_total_ is the steady-state cell current, p_0_ = 1.01 × 10^5^ Pa, R = 8.314 J mol^−1^ K^−1^.

**^15^N isotopic labeling experiment.** ^15^N_2_ (Wuhan Newradar Special Gas CO. LTD. 99 atom % ^15^N) was used as the feeding gas in the labeling experiment, and was sufficiently purified before use. After the electrolytic reaction, the obtained contained electrolyte was detected by ^1^H nuclear magnetic resonance measurement (Agilent 600 MHz, USA). (^15^NH_4_)_2_SO_4_ and (^14^NH_4_)_2_SO_4_ were used as the benchmark. The quantification of the produced ^15^NH_3_ was performed using the same method as that for ^14^NH_3_ either by colorimetric method or NMR spectra.

**Characterization after electrochemical test.** For the characterization of the catalyst after electrochemical nitrogen reduction test, continuous cyclic voltammetry (CV) scanning was first conducted at a potential range of -0.8~0.2 V vs. RHE in argon-saturated 0.1 M KOH electrolyte until the CV curves become stable, in order to clean up the residual nitrogenous intermediates. Then, the catalyst was carefully removed from the current collector and sufficiently washed for several times with plenty of water and ethanol under sonication. Finally, the catalyst was collected and heated at 120 °C under vacuum for at least 24 h before characterization.

**Computational method and model.** Cambridge Sequential Total Energy Package (CASTEP) was adopted to conduct the first principles calculations in the framework of density functional theory, including structural and electronic performances. The electron–electron interaction was described using the exchange-correlation functional under the generalized gradient approximation (GGA) with norm-conserving pseudopotentials and Perdew-Burke-Ernzerhof functional. 750 eV was used as the energy cutoff. Several parameters are considered, including energy tolerance of 5.0 × 10^-7^ eV per atom, a force tolerance of 0.01 eV Å^-1^ and maximum displacement of 5.0 × 10^-4^ Å. No constraints were set for any atom in the models, which is allowed to relax to the minimum in the enthalpy. The vacuum space along the z direction is set to be 15 Å.

Adsorption energy ΔE of A group on the surface of substrates was defined as:

| ΔE = E_*A_ – (E_*_+ E_A_) | (9) |
| --- | --- |

where *A and * denote the adsorption of A group on substrates and the bare substrates, E_A_ denotes the energy of A group.

Gibbs free energy change (ΔG) of each chemical reaction is calculated by:

| ΔG = ΔE + ΔZPE – TΔS + ΔG_U_ + ΔG_pH_ | (10) |
| --- | --- |

where E, ZPE, T, and S denote the calculated total energy, zero point energy, temperature, and entropy, respectively. ΔG_U_ = –eU (U is the potential measured against standard hydrogen electrode) and ΔG_pH_ = –kBTln(10)×pH. Here, T = 300 K and pH = 13 are considered.

The umbrella-sampling method was carried out to calculate the potential of mean force (PMF) for nitrogen adsorption towards the Co_4_-N_4_/C. The Co_4_-N_4_/C graphene sheet was initially placed perpendicular to the z-axis at the center of the simulation system, which contained 1 N_2_ molecule, 6 K^+^, 6 OH^-^, and 3000 water molecules.

Energy minimization (EM) by the steepest descent algorithm was first performed, followed by 1-ns isothermal-isobaric (NPT) equilibration at 298 K and 1 bar. Then the N_2_ molecule initially located at 1.5 nm above the geometric center of the upper three Co atoms was pulled downwards at a rate of 0.01 nm ps^–1^, after which 40 successive configurations were extracted, with the position of N_2_ molecule to be 0.03 nm apart in adjacent two configurations. Subsequently, umbrella sampling was performed for each of the above configurations for 8 ns at NPT ensemble (298 K, 1 bar), with system temperature and pressure controlled by Nose-Hoover thermostat and Parrinello-Rahman barostat, respectively.

For position restraints of the graphene sheet, a harmonic force constant of 10^4^ kJ mol^–1^ nm^–2^ was employed in all directions. With respect to the N_2_ molecule, three dimensional restraints with force constant of 10^6^ kJ mol^–1^ nm^–2^ was employed during EM and NPT equilibration, but only in the x-y plane during pulling and umbrella sampling. During pulling, a harmonic force constant of 10^5^ kJ mol^–1^ nm^–2^ was exerted at the N_2_ molecule along the z direction, while it was 10^4^ kJ mol^–1^ nm^–2^ during umbrella sampling. The PMF curve for N_2_ adsorption was obtained by processing the umbrella sampling trajectories with the weighted histogram analysis method (WHAM).





**Supplementary Figure 1.** TEM image of Co_SC_-N-C.


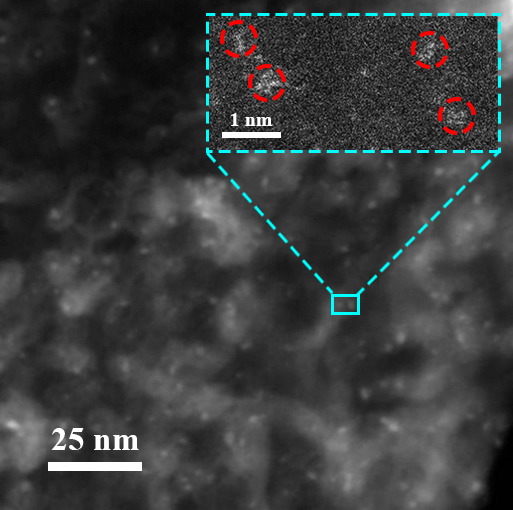


**Supplementary Figure 2.** Dark-field TEM image and HAADF-STEM image of Co_SC_-N-C.


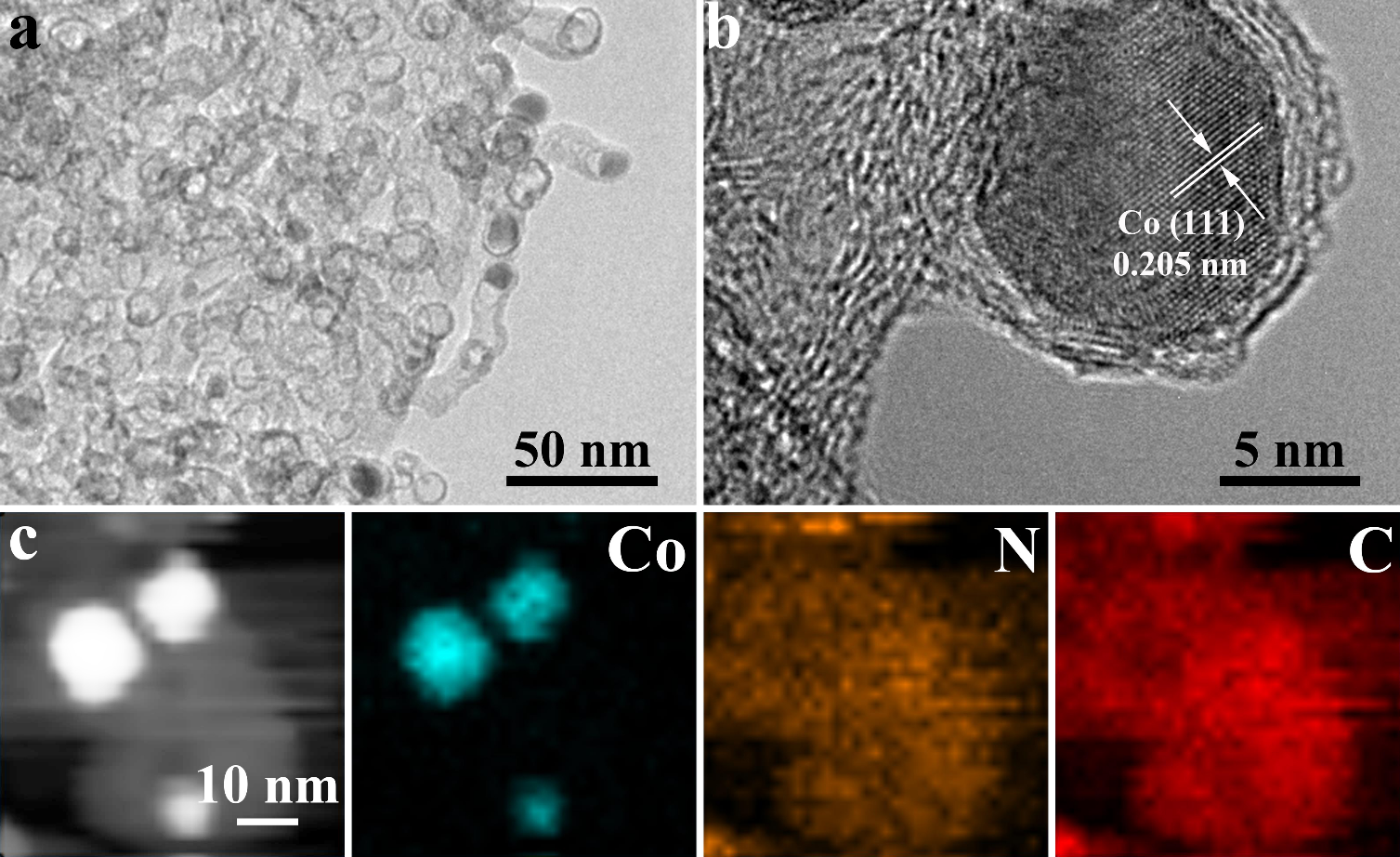


**Supplementary Figure 3.** (a) TEM image, (b) HRTEM image, and (c) corresponding element maps of Co_NP_-N-C.


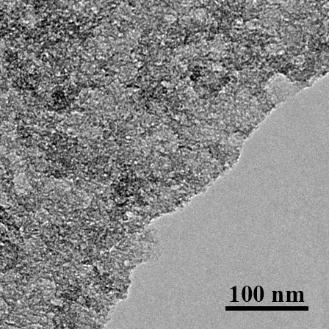


**Supplementary Figure 4.** TEM image of N-C.


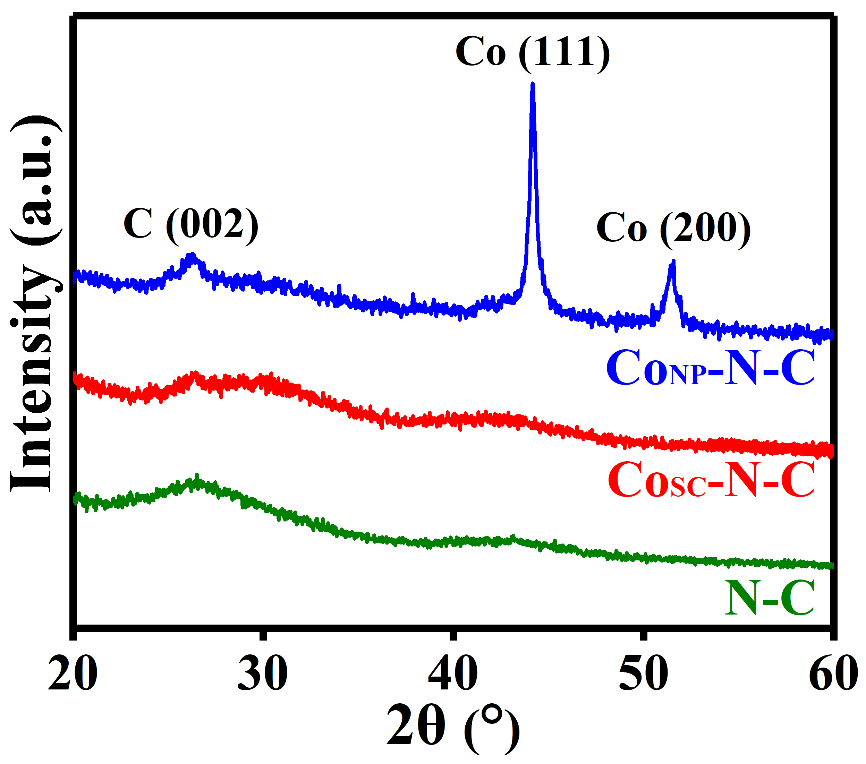


**Supplementary Figure 5.** XRD patterns of different samples.





**Supplementary Figure 6.** Raman spectra of different samples.





**Supplementary Figure 7.** The survey XPS spectra of different samples.


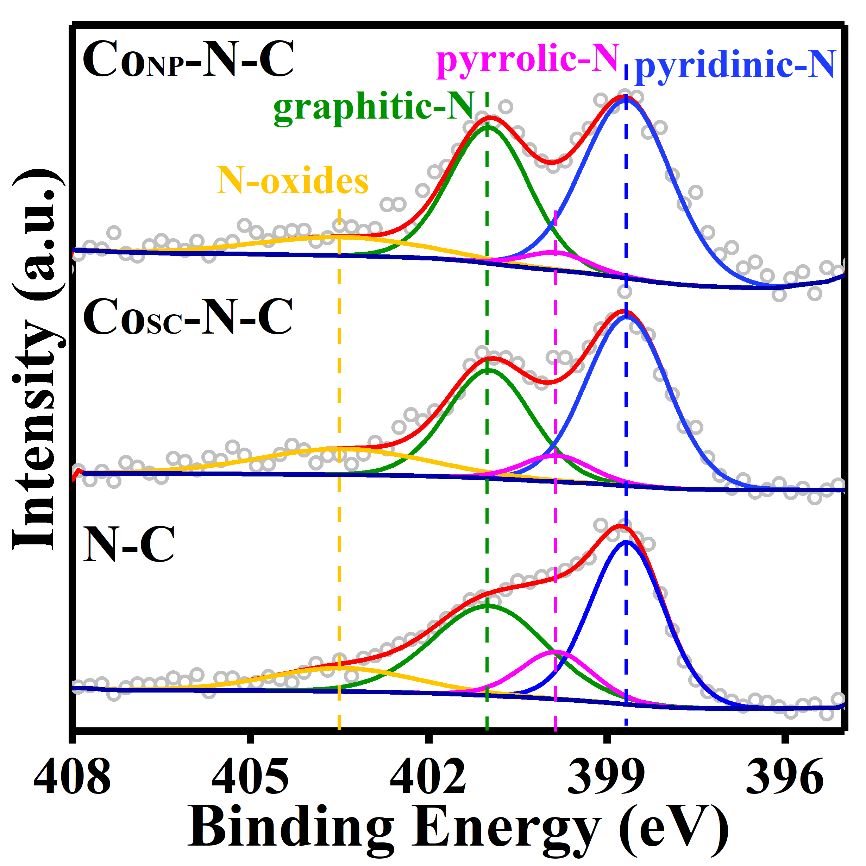


**Supplementary Figure 8.** High resolution N 1s spectra of different samples.


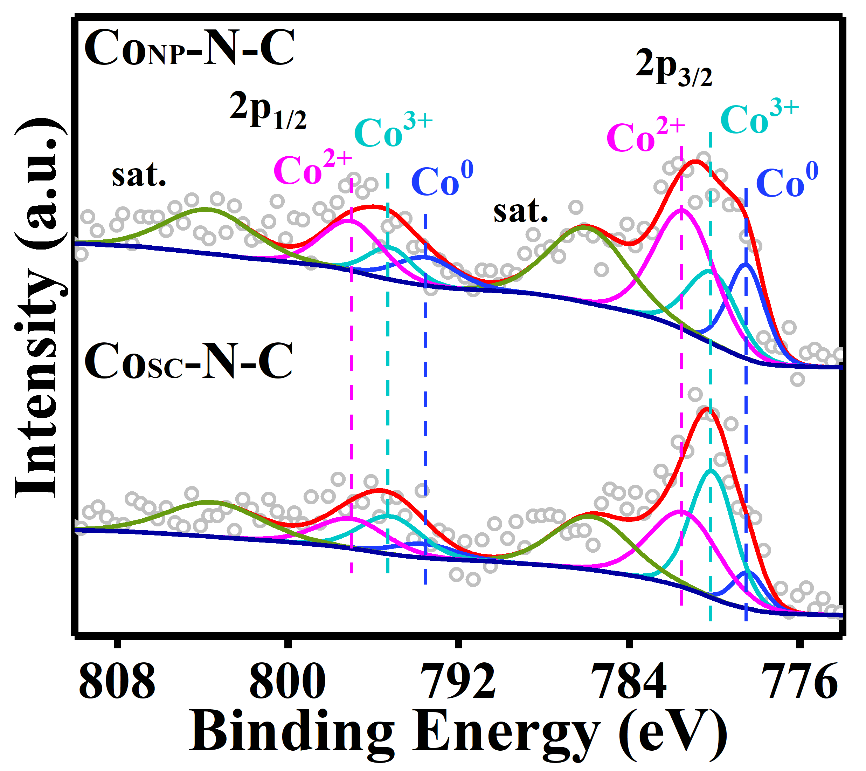


**Supplementary Figure 9.** High resolution Co 2p spectra of Co_NP_-N-C and Co_SC_-N-C.


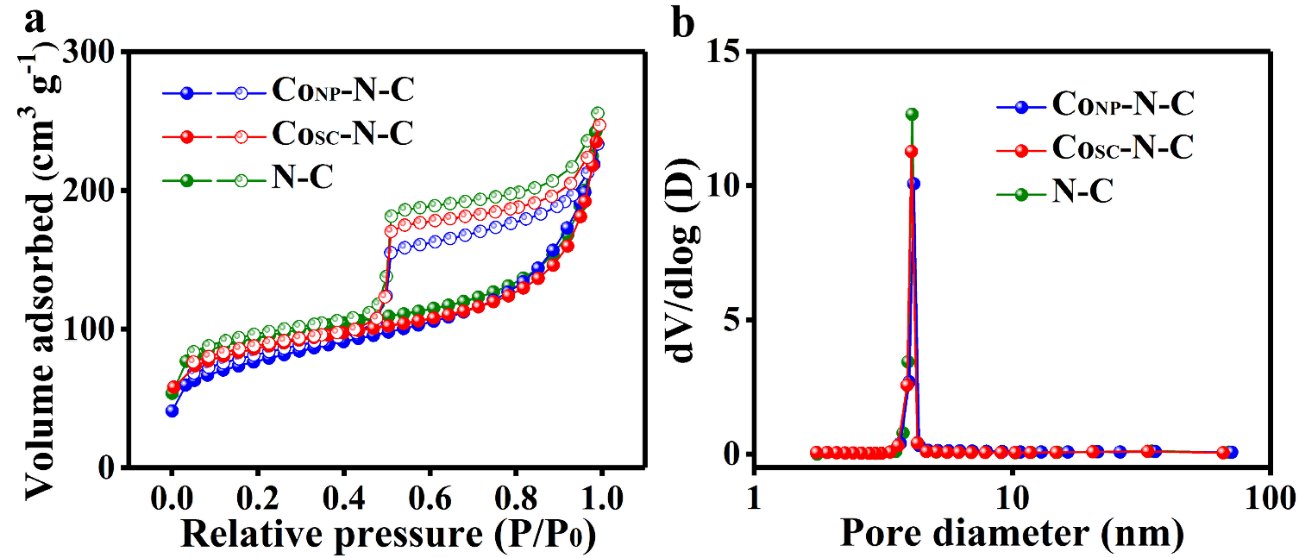


**Supplementary Figure 10.** (a) N_2_ adsorption/desorption isotherms and (b) the calculated pore size distribution curves of different samples. The catalysts possess large surface areas of 287.1 m^2^ g^−1^, 262.3 m^2^ g^−1^, and 311.6 m^2^ g^−1^ for Co_SC_-N-C, Co_NP_-N-C, and N-C, respectively, and exhibit similar pore size distribution.

**
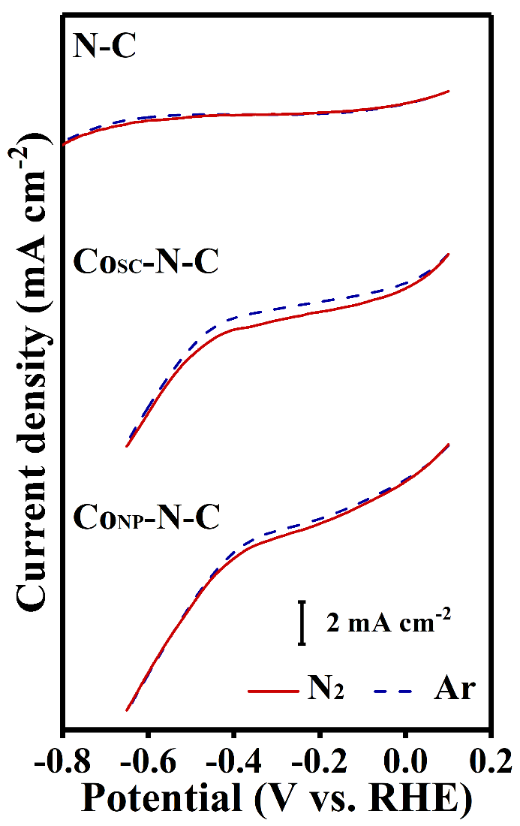
**

**Supplementary Figure 11.** Linear sweep voltammograms of N-C, Co_SC_-N-C, and Co_NP_-N-C in Ar-saturated (blue dashed line) or N_2_-saturated (red solid line) 0.1 M KOH solution with a scan rate of 50 mV s^−1^.


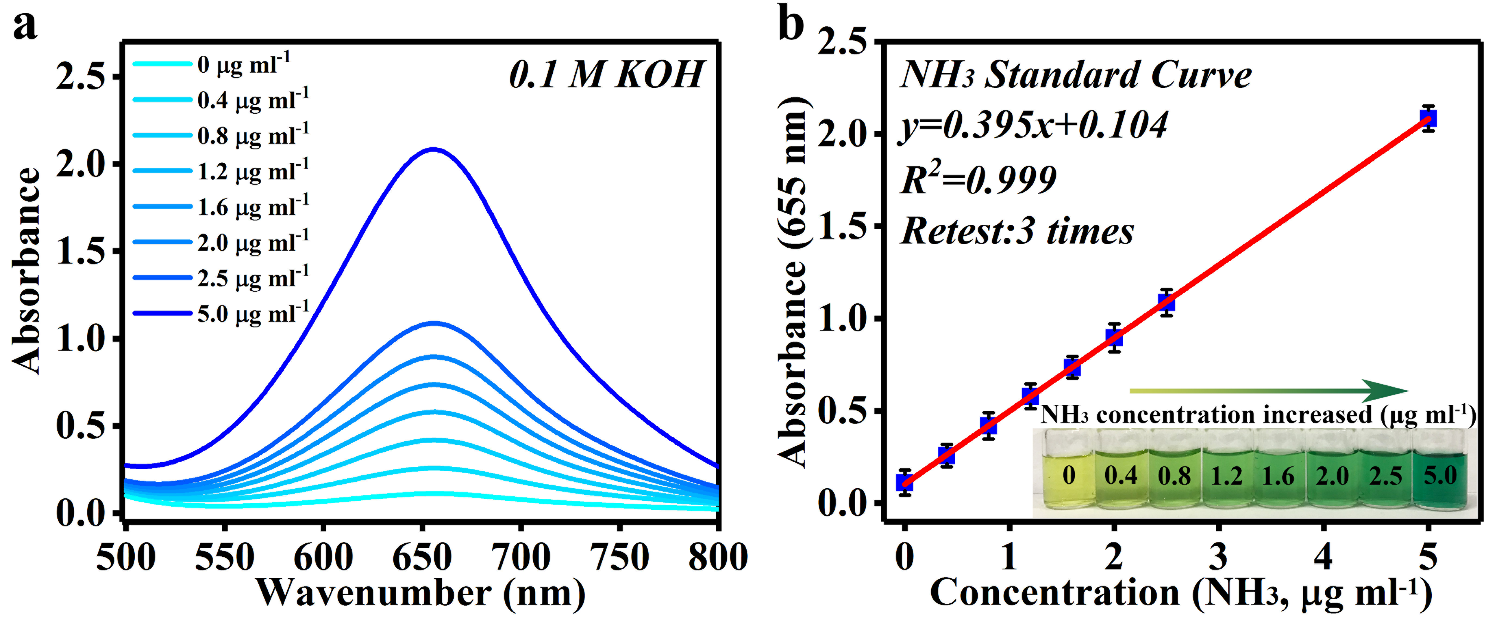


**Supplementary Figure 12.** (a) The UV-Vis absorption spectra and (b) corresponding calibration curves for the colorimetric NH_3_ assay using the indophenol blue method in 0.1 M KOH.


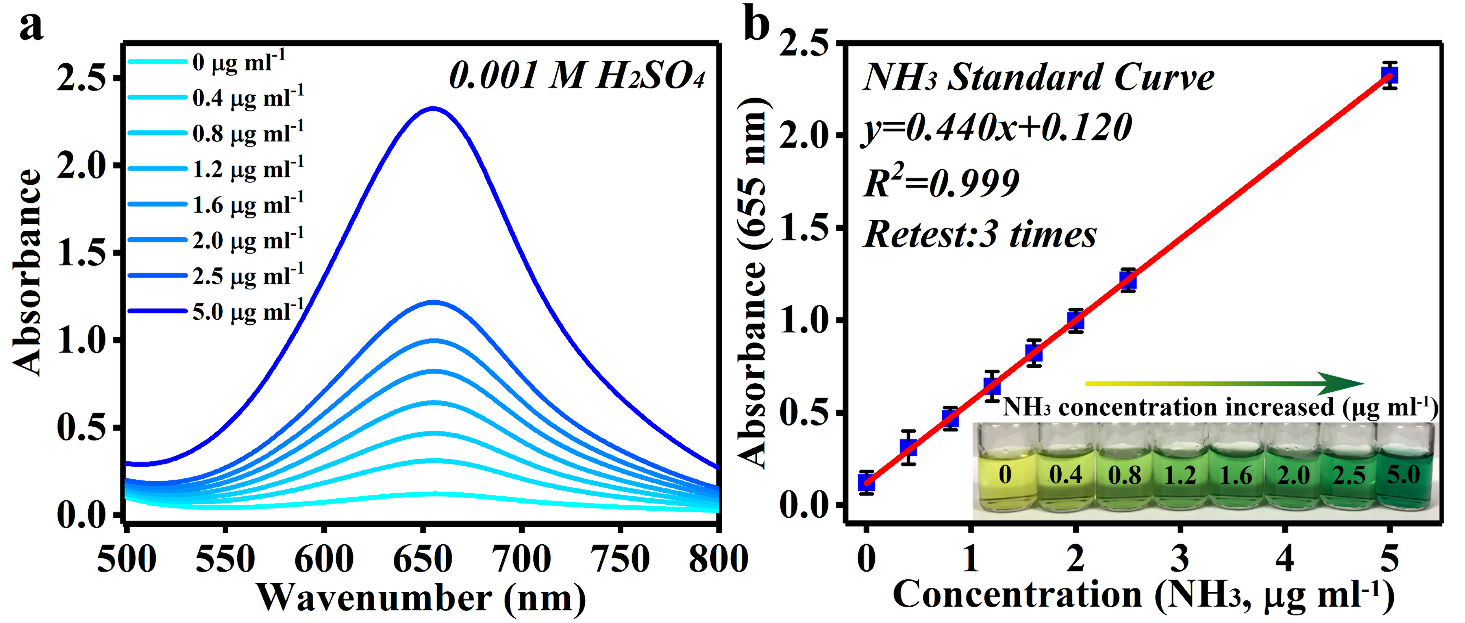


**Supplementary Figure 13.** (a) The UV-Vis absorption spectra and (b) corresponding calibration curves for the colorimetric NH_3_ assay using the indophenol blue method in 0.001 M H_2_SO_4_.


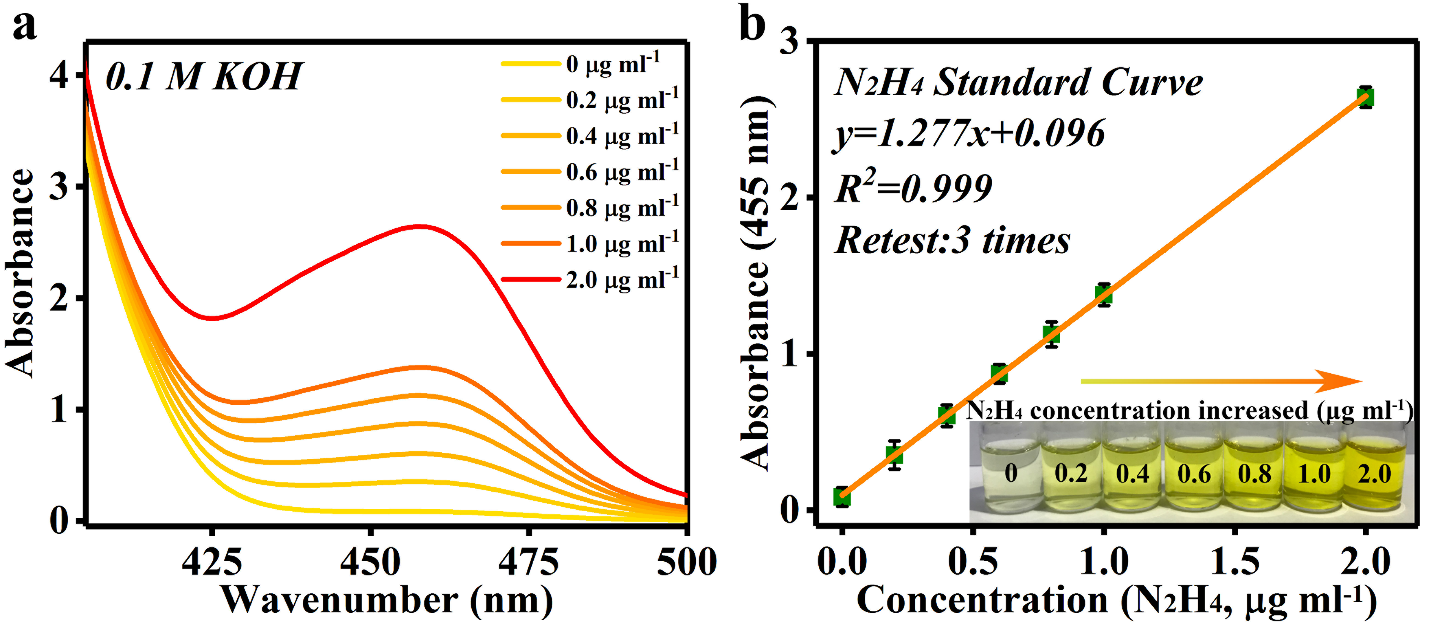


**Supplementary Figure 14.** (a) The UV-Vis absorption spectra and (b) corresponding calibration curves for the colorimetric N_2_H_4_ assay using the Watt and Chrisp method in 0.1 M KOH.


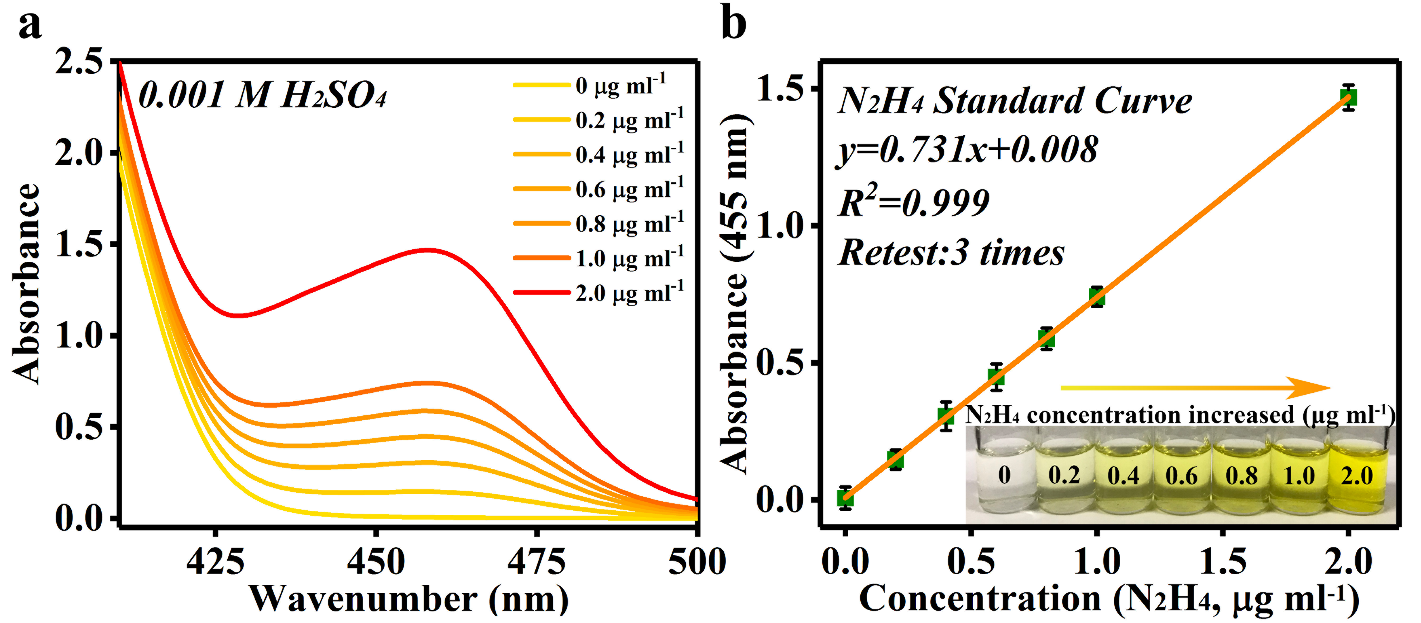


**Supplementary Figure 15.** (a) The UV-Vis absorption spectra and (b) corresponding calibration curves for the colorimetric N_2_H_4_ assay using the Watt and Chrisp method in 0.001 M H_2_SO_4_.


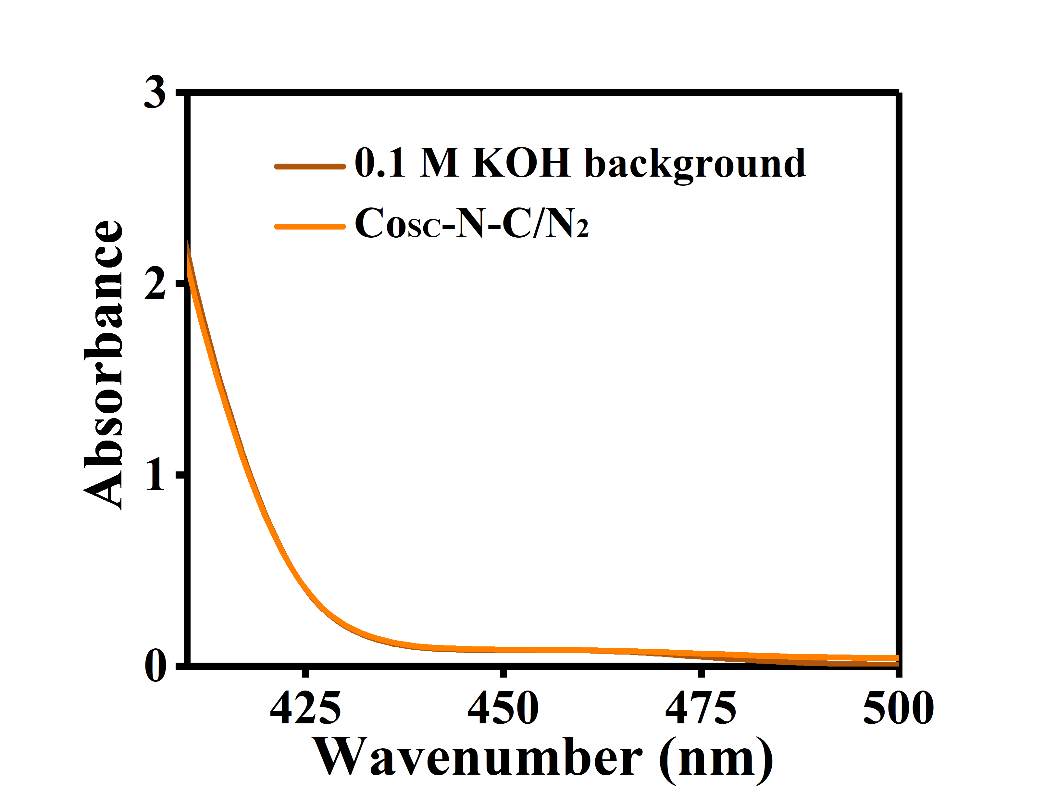


**Supplementary Figure 16.** The UV-Vis absorption spectra of the electrolytes estimated by the method of Watt and Chrisp. The result shows that no N_2_H_4_ was detected in this work.


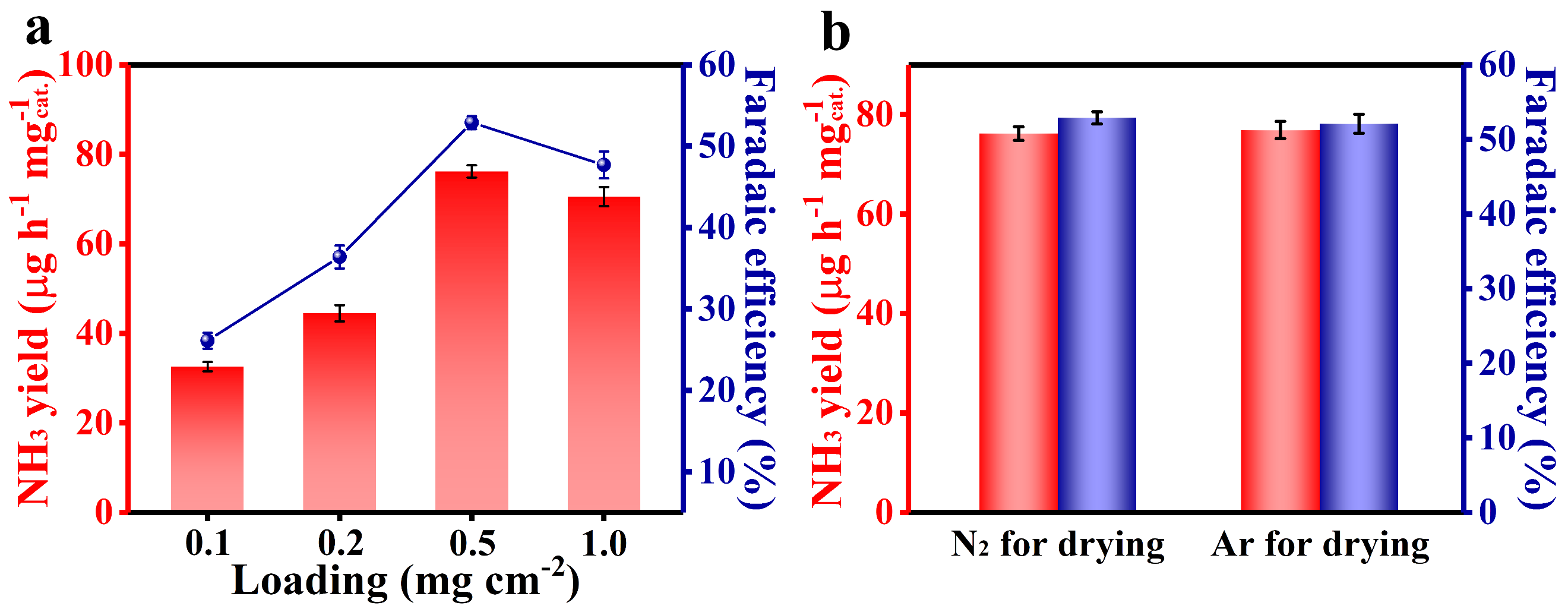


**Supplementary Figure 17.** (a) The NH_3_ yield and Faradaic efficiency of Co_SC_-N-C with different loadings at the potential of -0.2 V vs. RHE. (b) Comparison of the NRR performance of the Co_SC_-N-C electrodes obtained after drying under different gas atmospheres. The results show that the NH_3_ yield and Faradaic efficiency both reach the highest values at the catalyst loading of 0.5 mg cm^-2^, so that we accordingly optimized the catalyst loading as 0.5 mg cm^−2^ for the NRR tests. The gas atmosphere used for cathode drying would not cause any significant influence to the NRR performance.


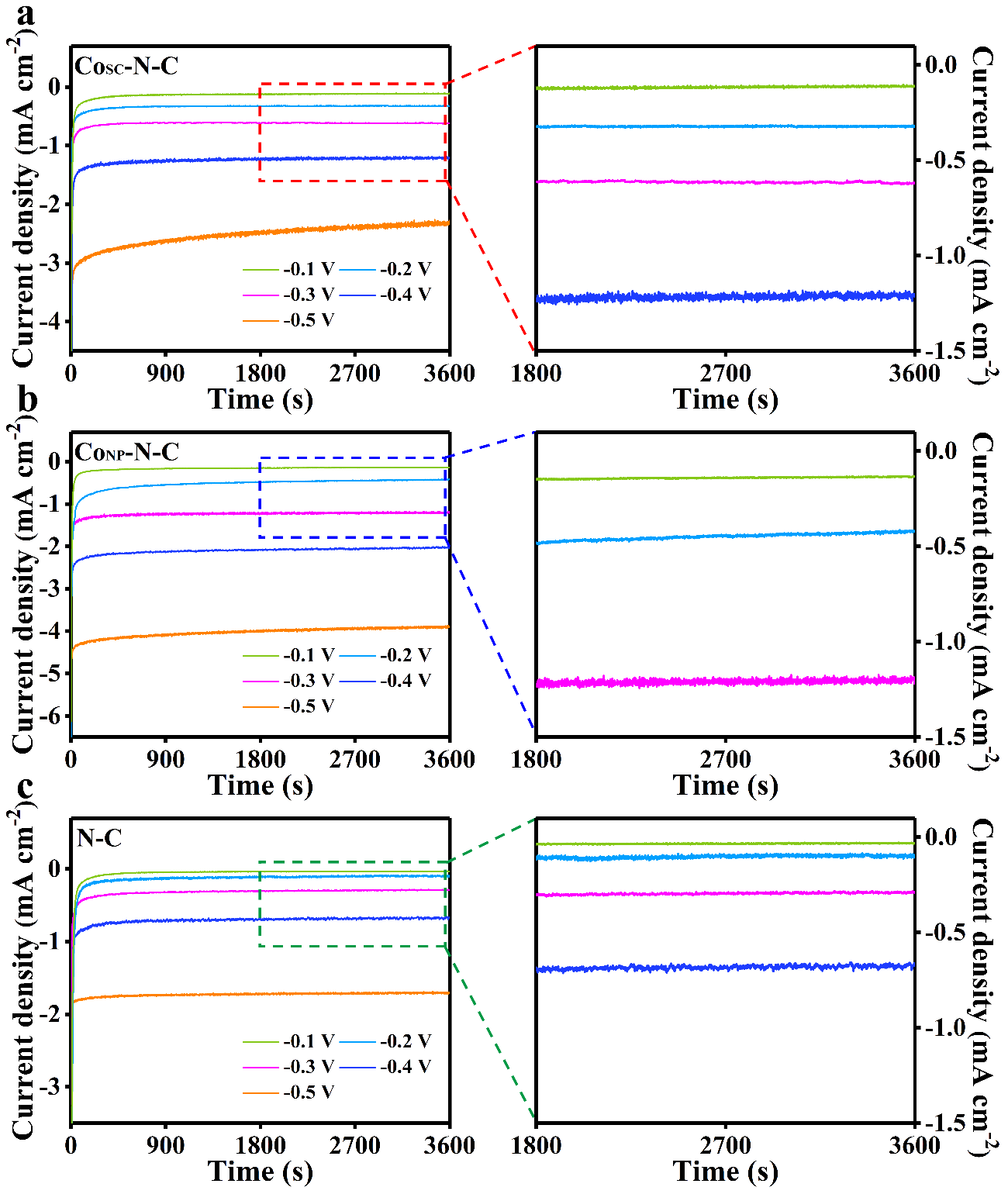


**Supplementary Figure 18.** Chronoamperometry results and the enlarged view of the curves of (a) Co_SC_-N-C, (b) Co_NP_-N-C, and (c) N-C at the corresponding potentials.


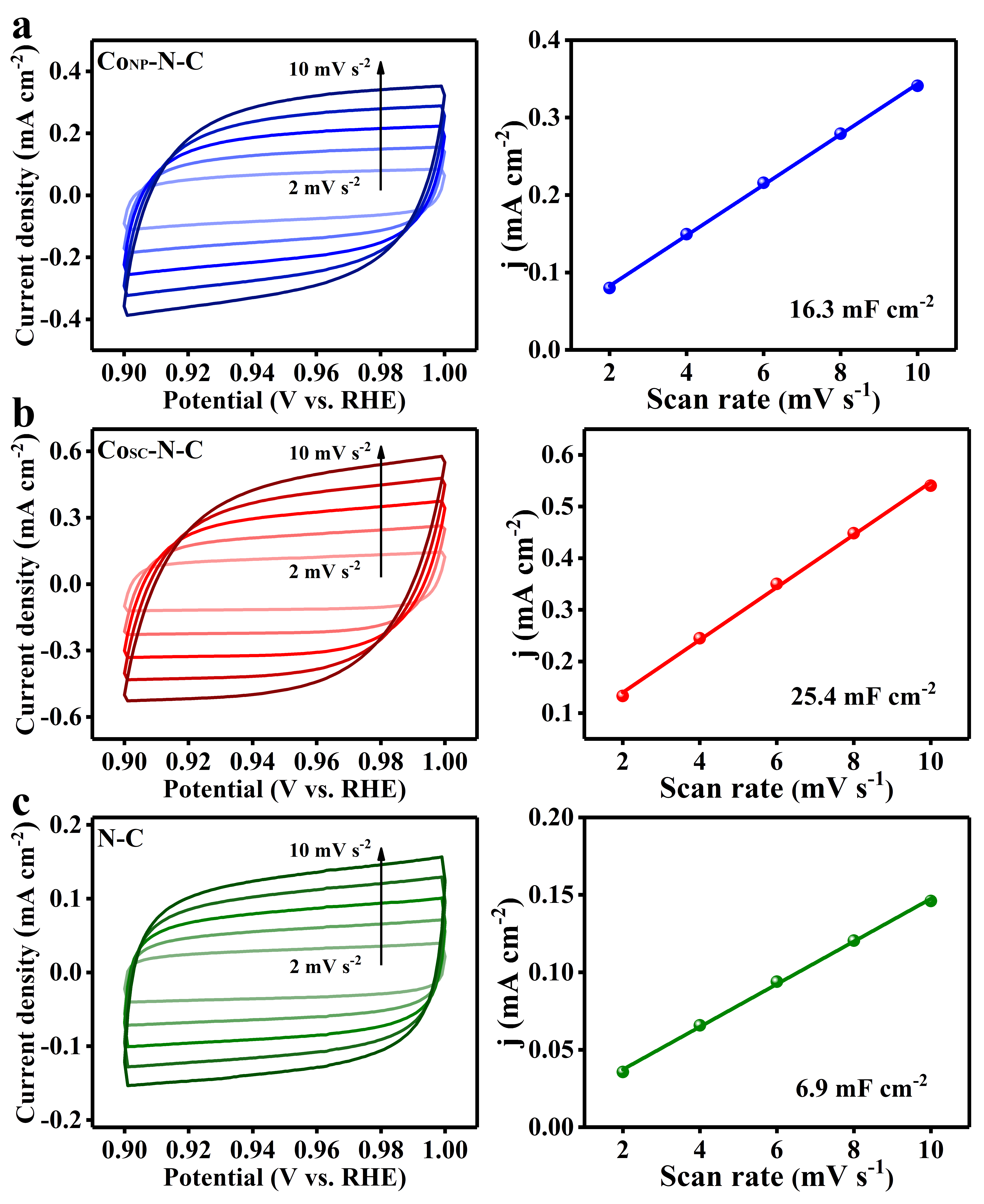


**Supplementary Figure 19.** Cyclic voltammetry curves measured at different scan rates from 2 to 10 mV s^-1^ and corresponding plots of the current density at 0.98 V vs. the scan rate of (a) Co_NP_-N-C, (b) Co_SC_-N-C, and (c) N-C for electrochemical double-layer capacitance (C_dl_) calculation. Since the electrochemical active surface area (EASA) is proportional to the C_dl_, the results show that the Co_SC_-N-C possess the largest EASA among the samples.


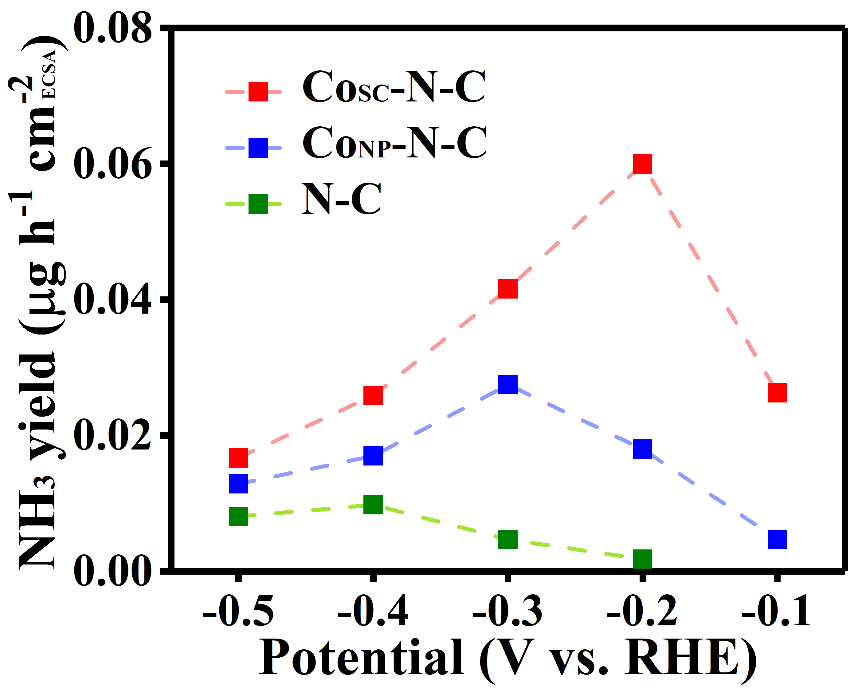


**Supplementary Figure 20.** Surface-area-normalized yield rate of NH_3_ production at different applied potentials of Co_SC_-N-C, Co_NP_-N-C, and N-C.

**Supplementary Table 1.** Comparison of the NRR performance of the Co_SC_-N-C catalyst with other catalysts reported to date under ambient conditions (room temperature and atmospheric pressure).

| Catalyst | Electrolyte | Potential  (V vs. RHE) | Faradaic efficiency (%) | NH_3_ yield rate  (μg h^–1^ mg–1 cat.) | Ref. |
| --- | --- | --- | --- | --- | --- |
| Cobalt single clusters dispersed in nitrogen-doped carbon | 0.1 M KOH | –0.2 | 52.9 | 76.2 | **This work** |
| Metal-free catalysts | | | | | |
| Boron carbide nanosheet | 0.1 M HCl | –0.75 | 15.95 | 26.57 | *S1* |
| Electrochemically excitated COF on nitrogen-doped carbon | 0.1 M KOH | –0.2 | 45.43 | 12.53 | *S2* |
| N-doped porous carbon | 0.05 M H_2_SO_4_ | –0.9 | 1.42 | 23.80 | *S3* |
| Metal-free polymeric carbon nitride | 0.1 M HCl | –0.2 | 11.59 | 8.09 | *S4* |
| Few-layer black phosphorus nanosheets | 0.01 M HCl | –0.6 | 5.07 | 20.87 | *S5* |
|  |  | –0.7 | 3.09 | 31.37 |  |
| S-doped carbon nanosphere | 0.1 M Na_2_SO_4_ | −0.4 | 7.47 | 19.07 | *S6* |
| Nitrogen-doped porous carbon-500 | 0.005 M H_2_SO_4_ | −0.4 | 9.98 | 22.27 | *S7* |
| Non-noble metal-based catalysts | | | | | |
| Polyimide decorated copper | 0.1 M KOH | –0.3 | 6.56 | 2.48 | *S8* |
|  |  | –0.4 | ~5.50 | 3.44 |  |
| Zr^4+^-doped anatase TiO_2_ | 0.1 M KOH | –0.45 | 17.30 | 8.90 | *S9* |
| Single-atom dispersed Fe–N–C | 0.1 M KOH | 0 | 56.55 | 7.48 | *S10* |
| Black phosphorus quantum dots on SnO_2–x_ nanotube | 0.1 M Na_2_SO_4_ | –0.4 | 14.60 | 48.87 | *S11* |
| Bi_4_V_2_O_11_/  CeO_2_ hybrid | 0.1 M HCl | –0.2 | 10.16 | 23.21 | *S12* |
| Bismuth nanocrystals supported on carbon black | 0.5 M K_2_SO_4_ (pH=3.5) | –0.6 | 66.00 | 3400 | *S13* |
| Cr_2_O_3_ hollow microsphere | 0.1 M Na_2_SO_4_ | –0.9 | 6.78 | 25.30 | *S14* |
| MoS_2_ | 0.1 M Li_2_SO_4_ (PH=3) | –0.2 | 9.81 | 43.4 | *S15* |
| Fe−N/C− carbon nanotube | 0.1 M KOH | –0.2 | 9.28 | 34.83 | *S16* |
| Fe_3_Mo_3_C embedded in the carbon nanosheets | 1 M KOH  (0.7 MPa) | 0.1 | 14.74 | – | *S17* |
|  |  | –0.025 | – | 1.36 |  |
| Noble metal-based catalysts | | | | | |
| Carbon black-supported Pd nanoparticle | 0.1 M PBS | 0.1 | 8.20 | 1.35 | *S18* |
| Au_6_/Ni bimetallic nanoparticle | 0.05 M H_2_SO_4_ | –0.14 | 67.80 | 7.4 | *S19* |
| Au nanorod | 0.1 M KOH | −0.2 | 3.88 | 1.65 | *S20* |
| Ru single atoms on nitrogen-doped carbon | 0.05 M H_2_SO_4_ | –0.2 | 29.6 | 120.90 | *S21* |
| Au cluster/TiO_2_ | 0.1 M HCl | −0.2 | 8.11 | 21.40 | *S22* |
| a-Au/CeO-RGO | 0.1 M HCl | −0.2 | 10.10 | 8.30 | *S23* |


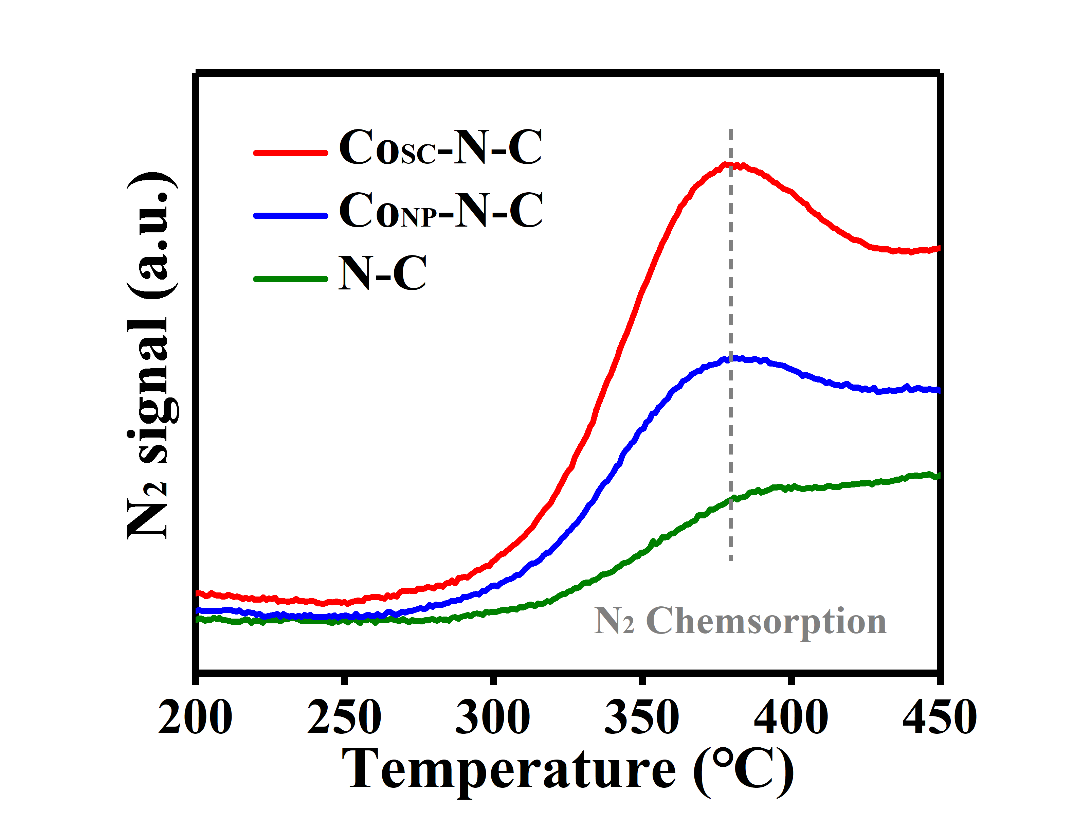


**Supplementary Figure 21.** N_2_-TPD spectra of different samples.


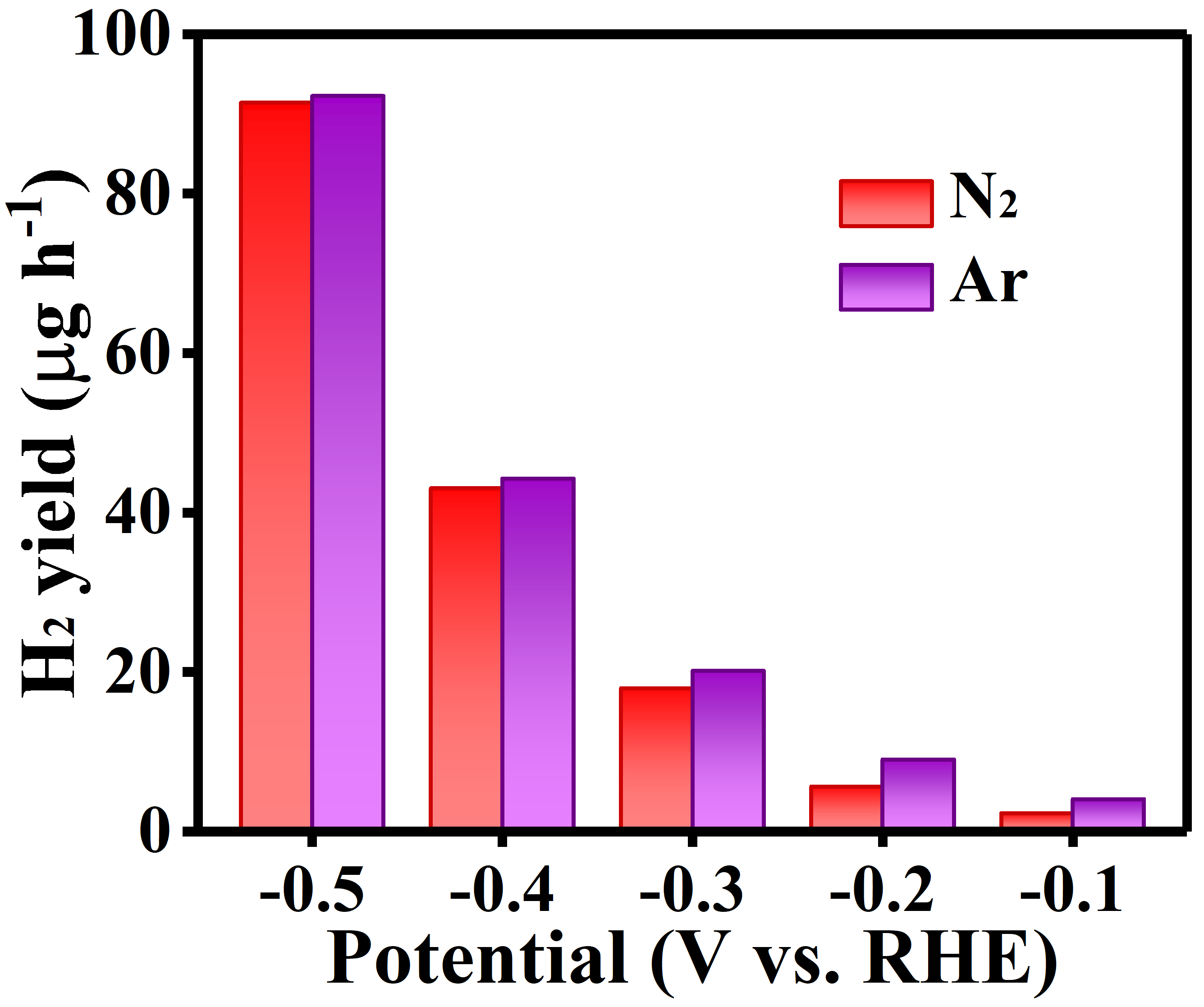


**Supplementary Figure 22.** The H_2_ yield of Co_SC_-N-C obtained by GC under N_2_ and Ar atmosphere.


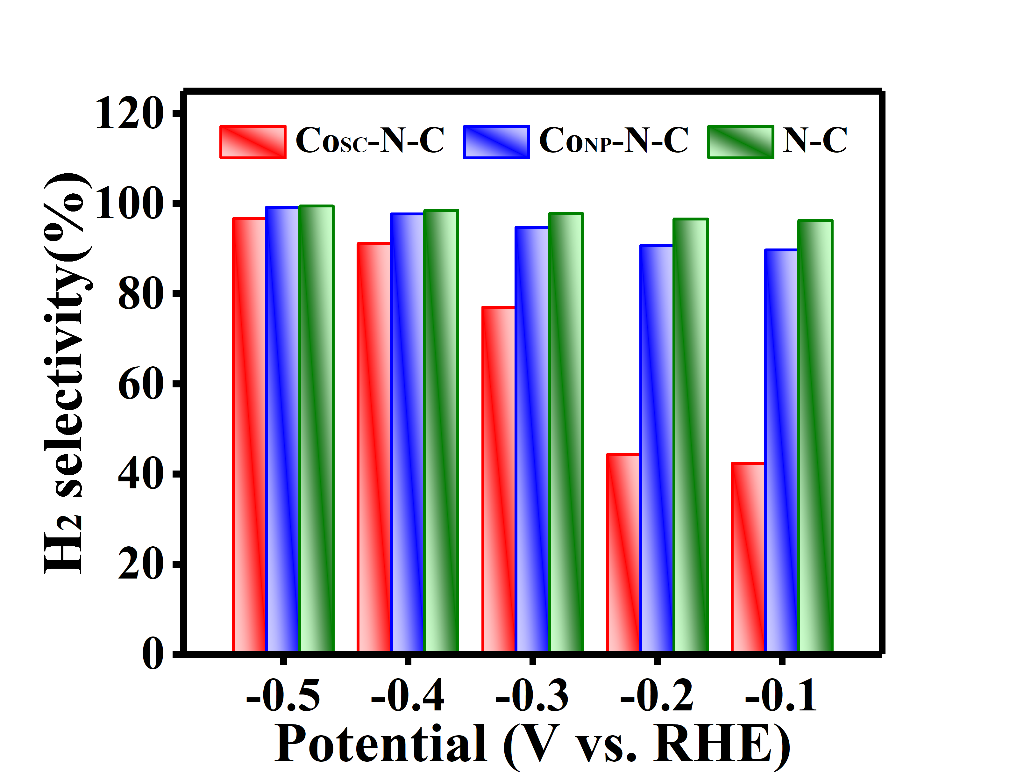


**Supplementary Figure 23.** The HER selectivity of different samples at each given potential under N_2_ atmosphere.

**Supplementary Table 2.** The performance of each sample under different applied potentials.

| Sample | Potential  (V vs. RHE) | NH_3_ yield rate  (μg h^–1^ mg–1 cat.) | NRR Faradaic efficiency (%) | HER Faradaic efficiency (%) |
| --- | --- | --- | --- | --- |
| Co_SC_-N-C | -0.1 | 31.3 | 50.7 | 42.4 |
|  | -0.2 | 76.2 | 52.9 | 44.4 |
|  | -0.3 | 52.8 | 20.0 | 77.1 |
|  | -0.4 | 32.9 | 6.2 | 91.2 |
|  | -0.5 | 21.2 | 2.0 | 96.7 |
| Co_NP_-N-C | -0.1 | 3.8 | 5.3 | 89.8 |
|  | -0.2 | 14.7 | 6.5 | 90.8 |
|  | -0.3 | 22.4 | 4.3 | 94.8 |
|  | -0.4 | 13.9 | 1.6 | 97.8 |
|  | -0.5 | 10.5 | 0.6 | 99.2 |
| N-C | -0.1 | - | - | 96.3 |
|  | -0.2 | 0.6 | 0.9 | 96.6 |
|  | -0.3 | 1.6 | 1.2 | 97.8 |
|  | -0.4 | 3.4 | 1.1 | 98.5 |
|  | -0.5 | 2.8 | 0.4 | 99.5 |


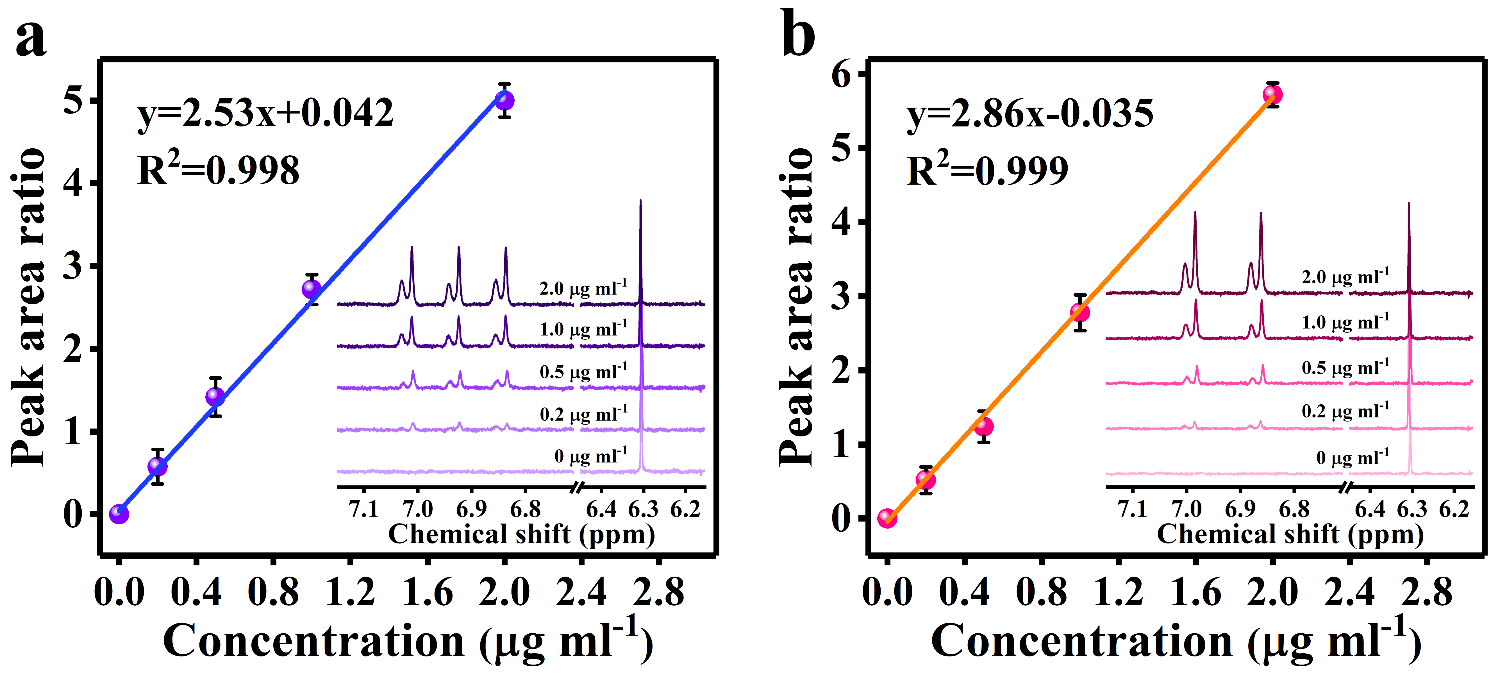


**Supplementary Figure 24.** Standard curve for (a) ^14^NH_4_^+^ and (b) ^15^NH_4_^+^ content vs. peak area ratio for NMR quantification method, with inset showing the corresponding NMR spectra of ^14^NH_4_^+^ and ^15^NH_4_^+^ with different normal concentrations.


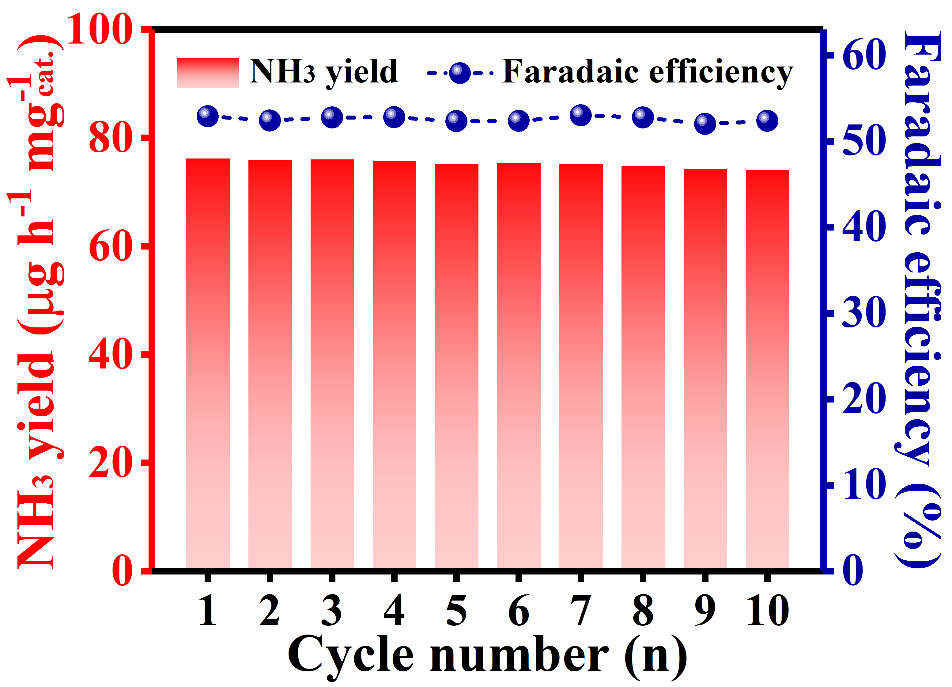


**Supplementary Figure 25.** NH_3_ yield rate and Faradaic efficiency of the Co_SC_-N-C catalyst obtained in the stability test.

**
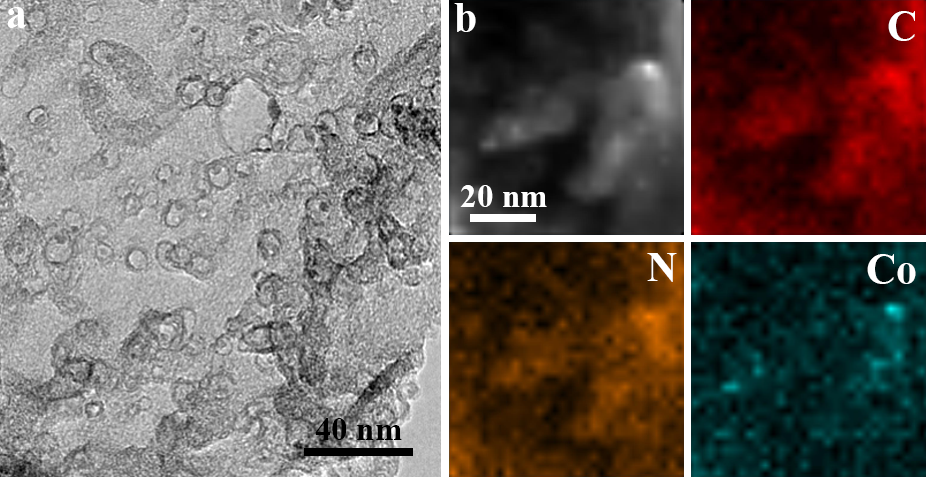
**

**Supplementary Figure 26.** (a) TEM image and (b) corresponding element maps of Co_SC_-N-C after NRR electrolysis.





**Supplementary Figure 27.** Raman spectra of Co_SC_-N-C before and after NRR electrolysis.

**
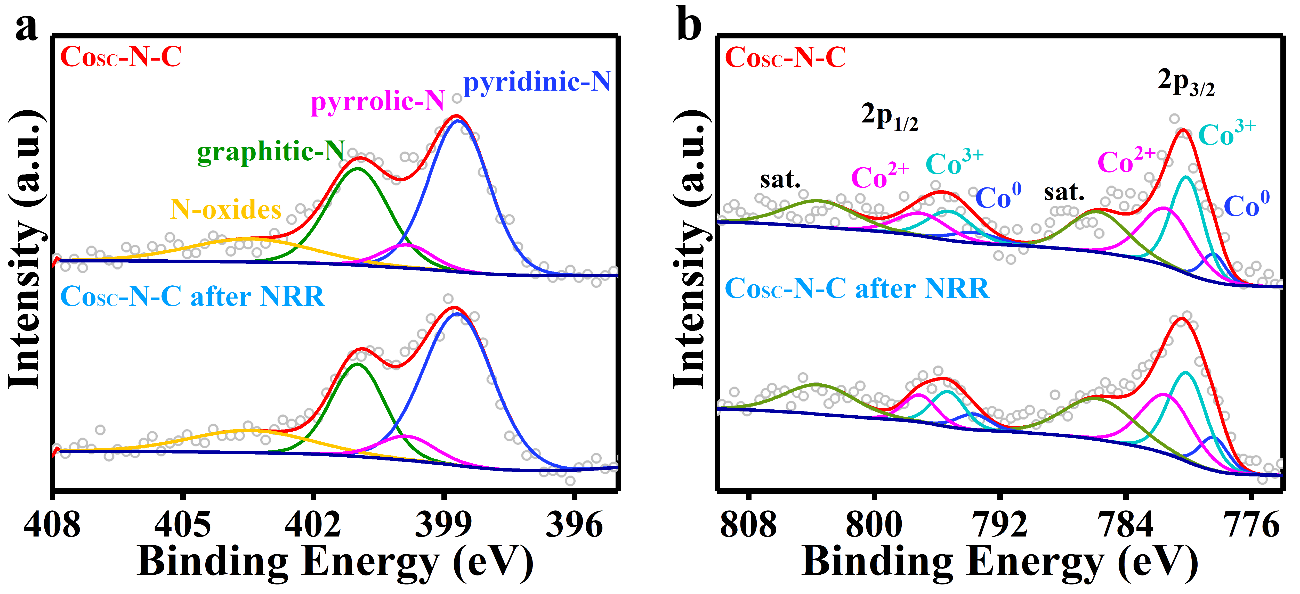
**

**Supplementary Figure 28.** High resolution N 1s and Co 2p spectra of Co_SC_-N-C before and after NRR electrolysis.

**
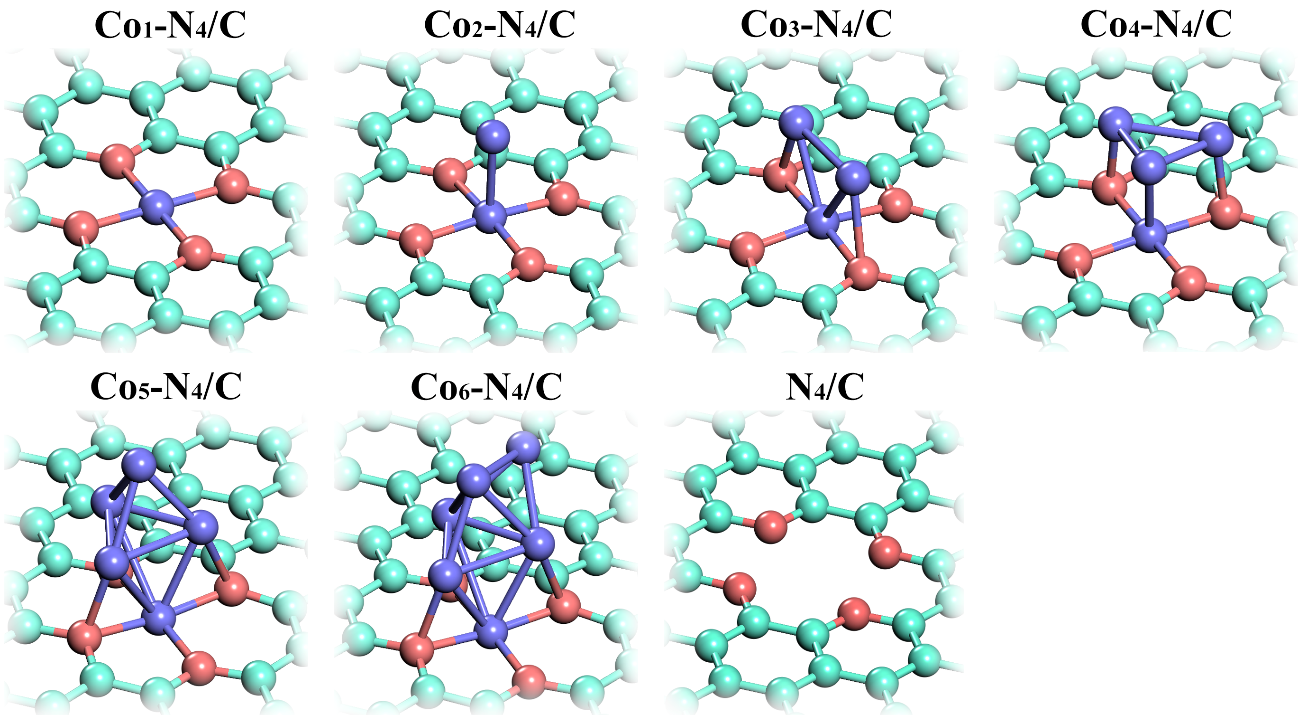
**

**Supplementary Figure 29.** Models for computational studies. The cyan, red, and purple spheres represent C, N, and Co atoms, respectively.


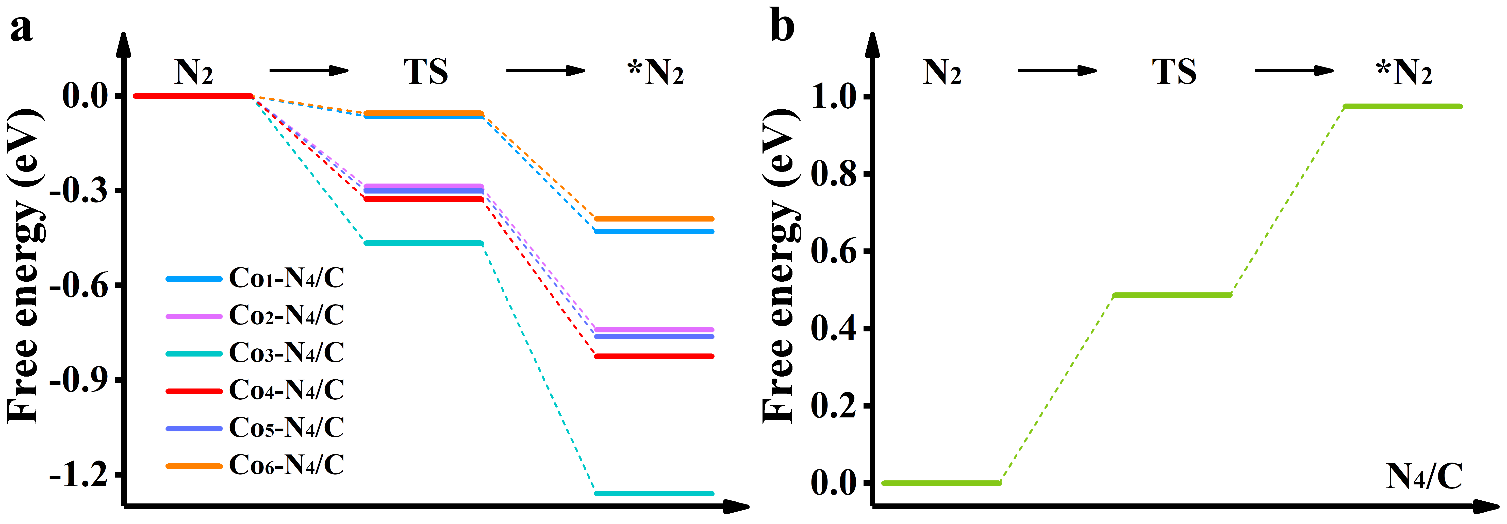


**Supplementary Figure 30.** Calculated free energy of the transition state (TS) of nitrogen chemisorption on (a) Co_x_-N_4_/C (x = 1 to 6) and (b) N_4_/C.


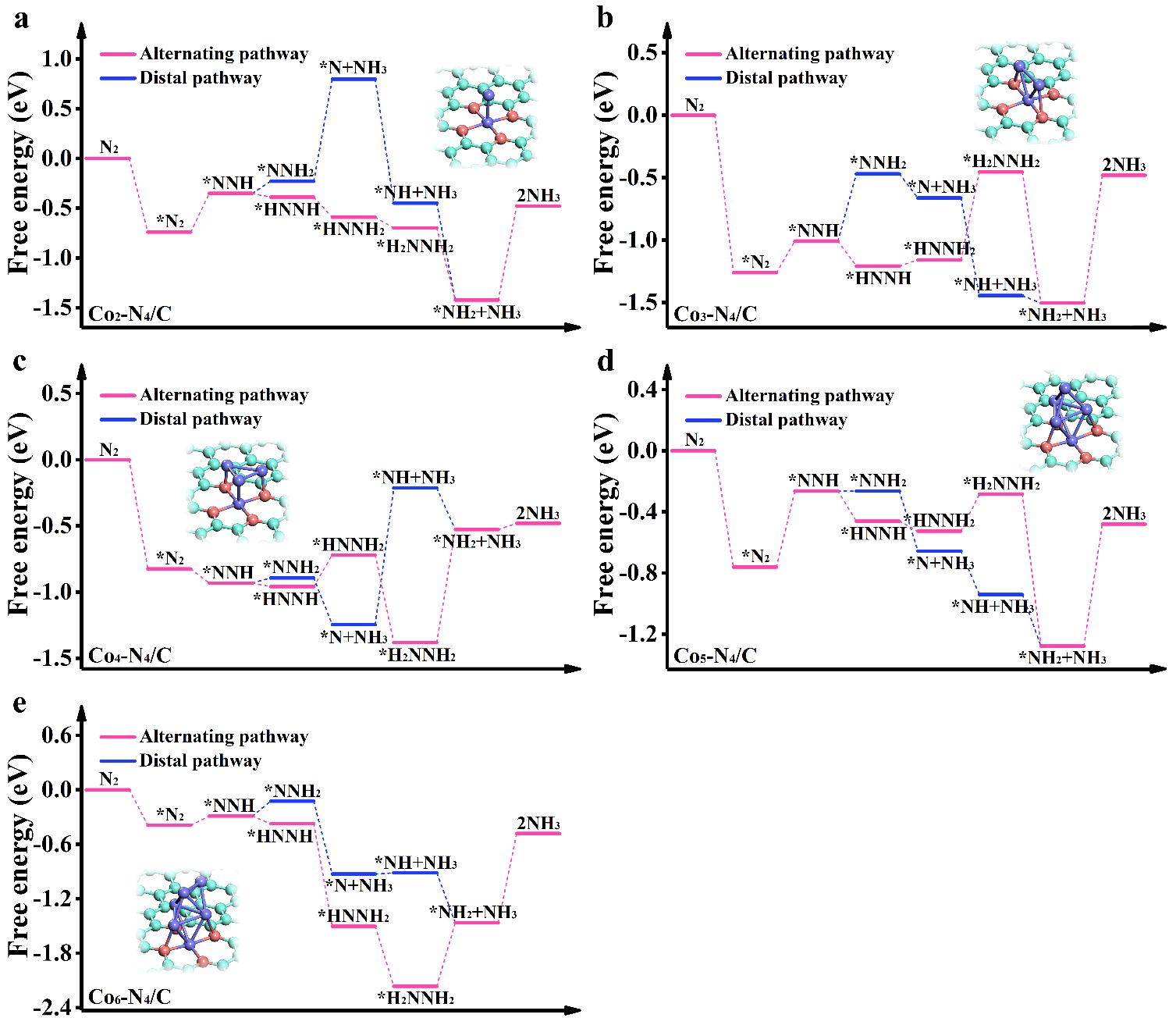


**Supplementary Figure 31.** Calculated free energy diagrams of (a) Co_2_-N_4_/C, (b) Co_3_-N_4_/C, (c) Co_4_-N_4_/C, (d) Co_5_-N_4_/C and (e) Co_6_-N_4_/C through the associative alternating pathway and the associative distal pathway. The cyan, red, and purple spheres represent C, N, and Co atoms, respectively. The results show that all of the structures with cobalt single cluster is able to alter the rate-determining step to the subsequent nitrogen hydrogenation.


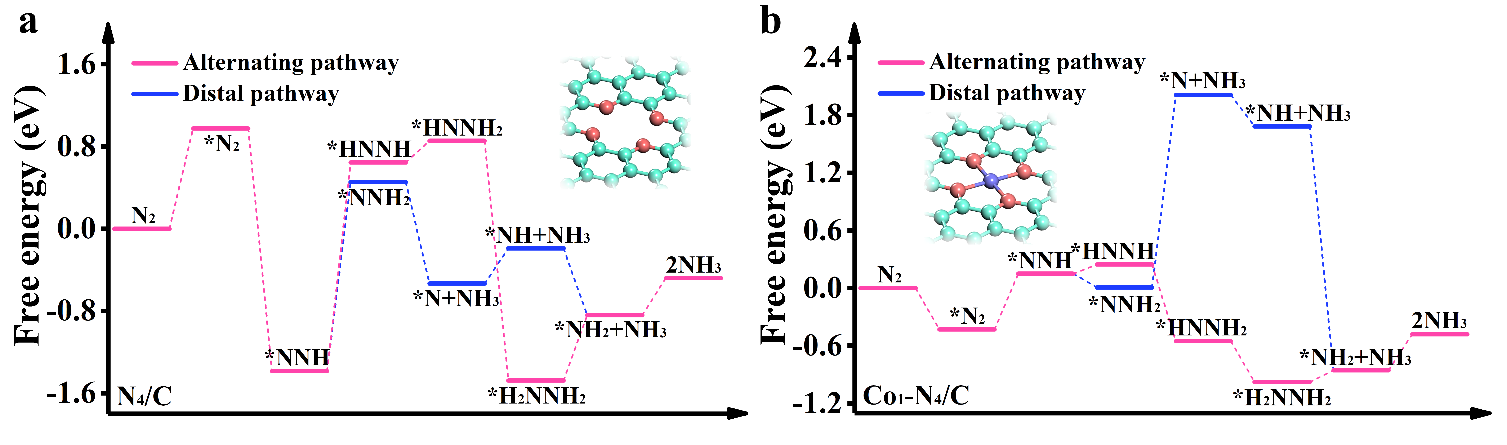


**Supplementary Figure 32.** Calculated free energy diagrams of (a) N_4_/C and (b) Co_1_-N_4_/C through the associative alternating pathway and the associative distal pathway. The cyan, red, and purple spheres represent C, N, and Co atoms, respectively.


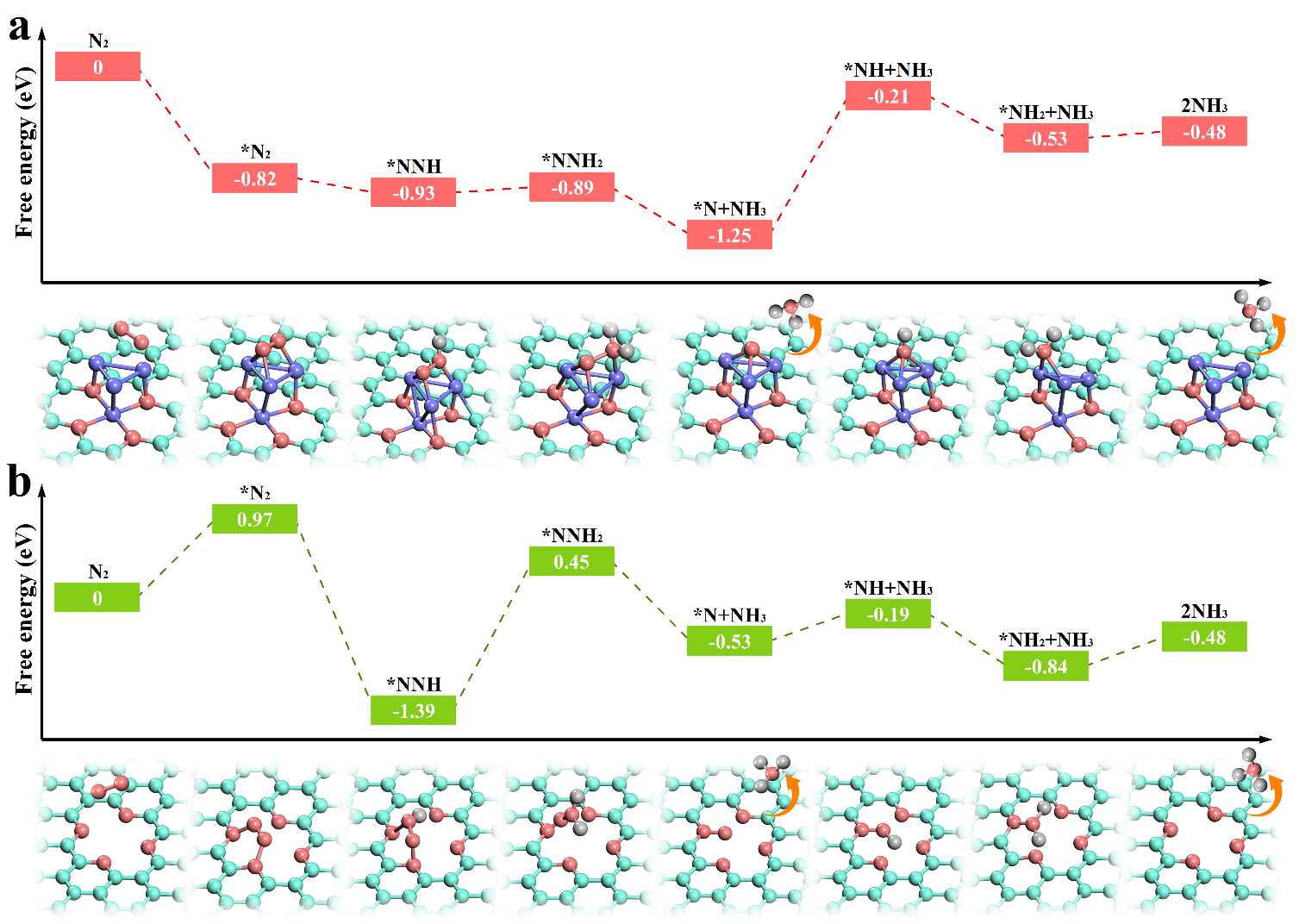


**Supplementary Figure 33.** Free energy diagram and models represent the corresponding adsorbates on (a) Co_4_-N_4_/C and (b) N_4_/C through associative distal pathway. The cyan, red, purple, and gray spheres represent C, N, Co, and H atoms, respectively.


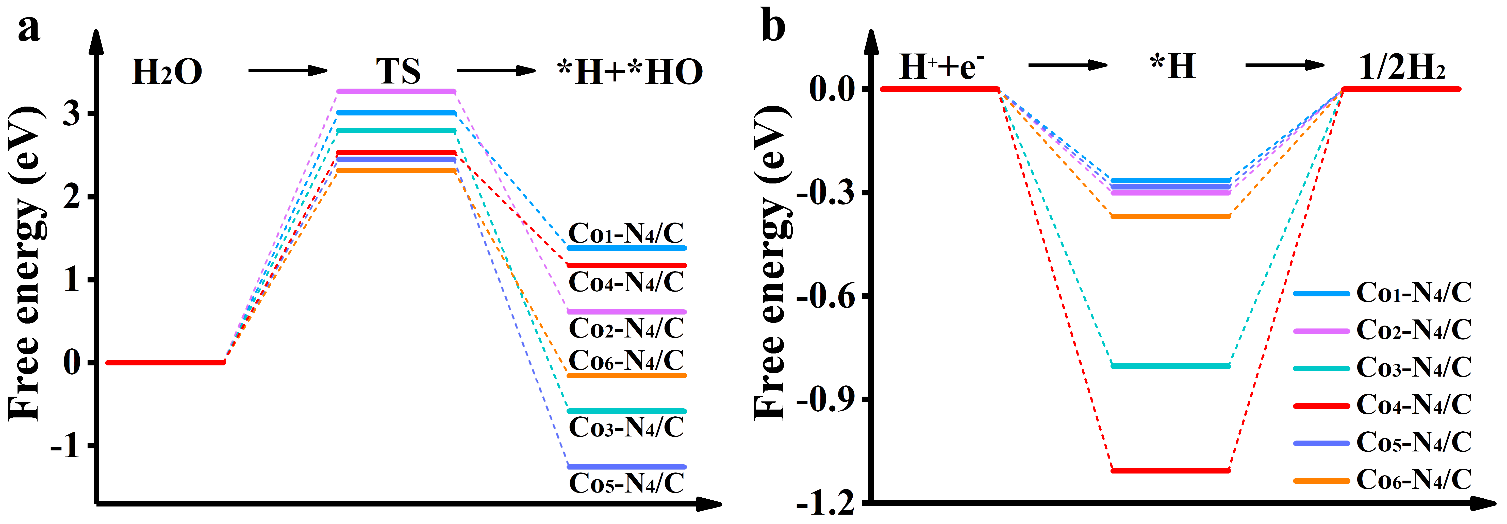


**Supplementary Figure 34.** Calculated free energy diagrams of (a) hydrogen chemisorption and (b) hydrogen evolution on different models.


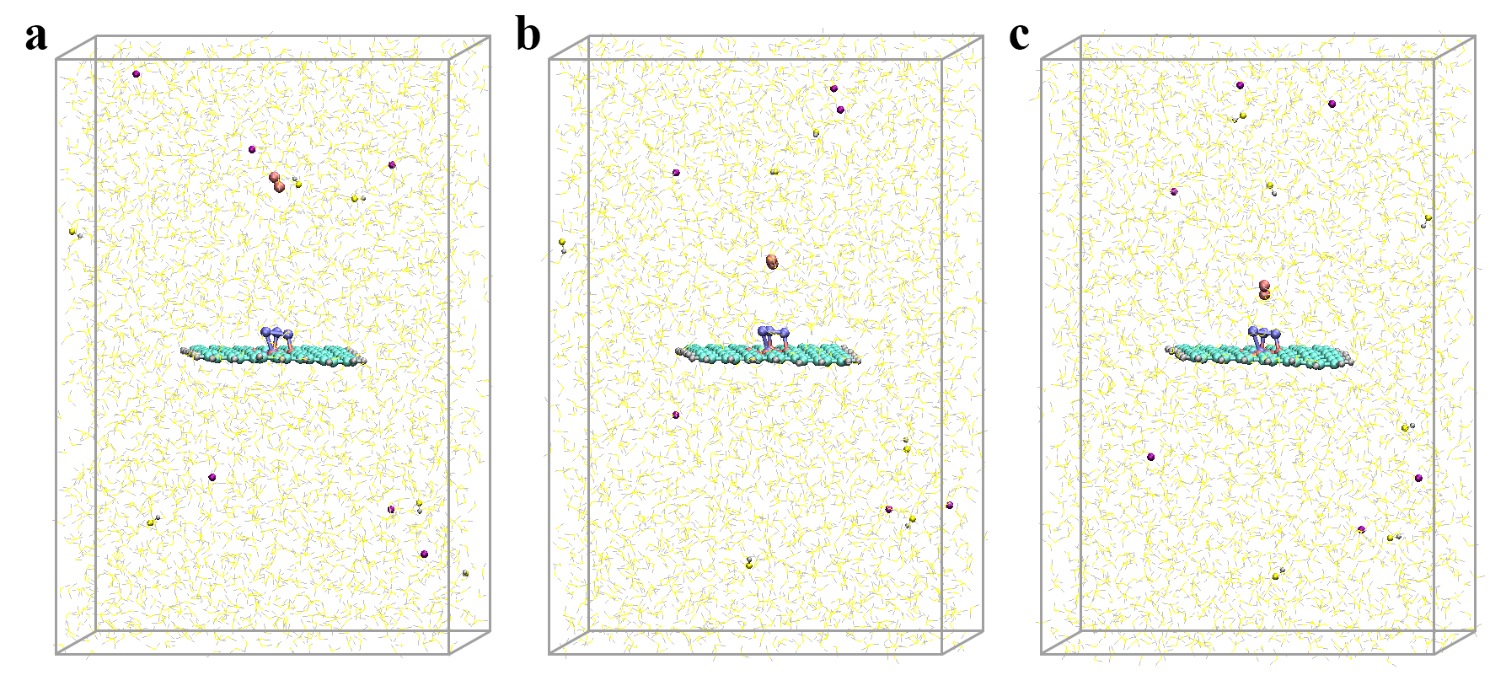


**Supplementary Figure 35.** MD simulation snapshots at (a) 1.50 nm, (b) 0.69 nm, and (c) 0.42 nm, with N_2_, red spheres; H_2_O, yellow and gray sticks; OH^−^, yellow and gray spheres; K^+^, wine red spheres.

**References**

S1. Qiu, W. et al. High-performance artificial nitrogen fixation at ambient conditions using a metal-free electrocatalyst. *Nat. Commun.***9**, 3485 (2018).

S2. Liu, S. et al. Facilitating nitrogen accessibility to boron-rich covalent organic frameworks via electrochemical excitation for efficient nitrogen fixation. *Nat. Commun.* **10**, 3898 (2019).

S3. Liu, Y. et al. Facile Ammonia Synthesis from Electrocatalytic N_2_ Reduction under Ambient Conditions on N‑Doped Porous Carbon. *ACS Catal.* **8**, 1186–1191 (2018).

S4. Lv, C. et al. Defect Engineering Metal-Free Polymeric Carbon Nitride Electrocatalyst for Effective Nitrogen Fixation under Ambient Conditions. *Angew. Chem. Int. Ed.* **57**, 10246–10250 (2018).

S5. Zhang, L., Ding, L. ­–X., Chen, G. ­–F., Yang, X. & Wang, H. Ammonia Synthesis Under Ambient Conditions: Selective Electroreduction of Dinitrogen to Ammonia on Black Phosphorus Nanosheets. *Angew. Chem. Int. Ed.* **58**, 2612–2616 (2019).

S6. Xia, L. et al. S-Doped Carbon Nanospheres: An Efficient Electrocatalyst toward Artificial N_2_ Fixation to NH_3_. *Small Methods* **2**, 1800251 (2018).

S7. Zhao, C. et al. Ambient Electrosynthesis of Ammonia on a Biomass-Derived Nitrogen-Doped Porous Carbon Electrocatalyst: Contribution of Pyridinic Nitrogen. *ACS Energy Lett.* **4**, 377−383 (2019).

S8. Lin, Y. –X. et al. Boosting selective nitrogen reduction to ammonia on electron-deficient copper nanoparticles. *Nat. Commun.* **10**, 4380 (2019).

S9. Cao, N. et al. Doping strain induced bi-Ti^3+^ pairs for efficient N_2_ activation and electrocatalytic fixation. *Nat. Commun.* **10**, 2877 (2019).

S10. Wang, M. et al. Over 56.55% Faradaic efficiency of ambient ammonia synthesis enabled by positively shifting the reaction potential. *Nat. Commun.* **10**, 341 (2019).

S11. Ding, B., Liu, Y. –T., Li, D. & Yu, J. Stable Confinement of Black Phosphorus Quantum Dots on Black Tin Oxide Nanotubes: A Robust, Double-Active Electrocatalyst toward Efficient Nitrogen Fixation. *Angew. Chem. Int. Ed.* 10.1002/anie.201908415 (2019).

S12. Lv, C. et al. An Amorphous Noble-Metal-Free Electrocatalyst that Enables Nitrogen Fixation under Ambient Conditions. *Angew. Chem. Int. Ed.* **57**, 6073–6076 (2018).

S13. Hao, Y. –C. et al. Promoting nitrogen electroreduction to ammonia with bismuth nanocrystals and potassium cations in water. *Nat. Catal.* **2**, 448–456 (2019).

S14. Zhang, Y. et al. High-Performance Electrohydrogenation of N_2_ to NH_3_ Catalyzed by Multishelled Hollow Cr_2_O_3_ Microspheres under Ambient Conditions. *ACS Catal.* **8**, 8540–8544 (2018).

S15. Liu, Y. et al. Dramatically Enhanced Ambient Ammonia Electrosynthesis Performance by In-Operando Created Li–S Interactions on MoS_2_ Electrocatalyst. *Adv. Energy Mater.* **9**, 1803935 (2019).

S16. Wang, Y. et al. Rational Design of Fe−N/C Hybrid for Enhanced Nitrogen Reduction Electrocatalysis under Ambient Conditions in Aqueous Solution. *ACS Catal.* **9**, 336−344 (2019).

S17. Cheng, H., Cui, P., Wang, F., Ding, L. –X. & Wang, H. High Efficiency Electrochemical Nitrogen Fixation Achieved on a Low-Pressure Reaction System by Changing Chemical Equilibrium. *Angew. Chem. Int. Ed.* 10.1002/anie.201910658 (2019).

S18. Wang, J. et al. Ambient ammonia synthesis via palladium-catalyzed electrohydrogenation of dinitrogen at low overpotential. *Nat. Commun.* **9**, 1795 (2018).

S19. Xue, Z. ­­–H. et al. Electrochemical Reduction of N_2_ into NH_3_ by Donor−Acceptor Couples of Ni and Au Nanoparticles with a 67.8% Faradaic Efficiency. *J. Am. Chem. Soc.* **141**, 14976−14980 (2019).

S20. Bao, D. et al. Electrochemical Reduction of N_2_ under Ambient Conditions for Artificial N_2_ Fixation and Renewable Energy Storage Using N_2_/NH_3_ Cycle. *Adv. Mater.* **29**, 1604799 (2017).

S21. Geng, Z. et al. Achieving a Record-High Yield Rate of 120.9 μg mg^–1^ h^–1^ for N_2_ Electrochemical Reduction over Ru Single-Atom Catalysts. *Adv. Mater.* **30**, 1803498 (2018).

S22. Shi, M. –M. et al. Au Sub-Nanoclusters on TiO_2_ toward Highly Efficient and Selective Electrocatalyst for N_2_ Conversion to NH_3_ at Ambient Conditions. *Adv. Mater.* **29**, 1606550 (2017).

S23. Li, S. –J. et al. Amorphizing of Au Nanoparticles by CeO_x_–RGO Hybrid Support towards Highly Efficient Electrocatalyst for N_2_ Reduction under Ambient Conditions. *Adv. Mater.* **29**, 1700001 (2017).
